# The Need for Accurate Osmotic Pressure and Mass Transfer Resistances in Modeling Osmotically Driven Membrane Processes

**DOI:** 10.3390/membranes11020128

**Published:** 2021-02-14

**Authors:** Endre Nagy, Imre Hegedüs, Danyal Rehman, Quantum J. Wei, Yvana D. Ahdab, John H. Lienhard

**Affiliations:** 1 Chemical and Biochemical Procedures Laboratory, Institute of Biomolecular and Chemical Engineering, University of Pannonia, H-8200 Veszprem, Hungary; hegedus@mukki.richem.hu; 2 Department of Biophysics and Radiation Biology, Semmelweis University, Tüzoltó u. 37-47, H-1094 Budapest, Hungary; 3 Rohsenow Kendall Heat Transfer Laboratory, Department of Mechanical Engineering, Massachusetts Institute of Technology, Cambridge, MA 02139, USA; drehman@mit.edu (D.R.); quantum.wei@gmail.com (Q.J.W.); yahdab@mit.edu (Y.D.A.)

**Keywords:** pressure-retarded osmosis, mass transport, osmotic pressure, van ’t Hoff approach, OLI software, power density, Porifera membrane, NanoH_2_O membrane

## Abstract

The widely used van ’t Hoff linear relation for predicting the osmotic pressure of NaCl solutions may result in errors in the evaluation of key system parameters, which depend on osmotic pressure, in pressure-retarded osmosis and forward osmosis. In this paper, the linear van ’t Hoff approach is compared to the solutions using OLI Stream Analyzer, which gives the real osmotic pressure values. Various dilutions of NaCl solutions, including the lower solute concentrations typical of river water, are considered. Our results indicate that the disparity in the predicted osmotic pressure of the two considered methods can reach 30%, depending on the solute concentration, while that in the predicted power density can exceed over 50%. New experimental results are obtained for NanoH2O and Porifera membranes, and theoretical equations are also developed. Results show that discrepancies arise when using the van ’t Hoff equation, compared to the OLI method. At higher NaCl concentrations (C > 1.5 M), the deviation between the linear approach and the real values increases gradually, likely indicative of a larger error in van ’t Hoff predictions. The difference in structural parameter values predicted by the two evaluation methods is also significant; it can exceed the typical 50–70% range, depending on the operating conditions. We find that the external mass transfer coefficients should be considered in the evaluation of the structural parameter in order to avoid overestimating its value. Consequently, measured water flux and predicted structural parameter values from our own and literature measurements are recalculated with the OLI software to account for external mass transfer coefficients.

## 1. Introduction

The osmotically driven membrane separation processes, such as pressure-retarded osmosis (PRO) and forward osmosis (FO), play a critical role in energy generation [1,2,3], water purification, and dewatering/desalination [4,5,6,7]. Consequently, these processes have been investigated experimentally [1,8,9], theoretically [10,11,12,13,14], and numerically [15,16,17] in the literature for aqueous sodium chloride solutions. Intensive research has also been carried out by hybrid PRO processes that combine reverse osmosis desalination and pressure-retarded osmosis [17,18,19,20] in order to reduce the energy consumption of water desalination. The osmotic pressure with brine solution as draw [19,21] and wastewater retentate as feed [22] has also been studied. With the increasing application of hollow-fiber modules, several studies have numerically evaluated and optimized scaled-up modules [15,16,23,24,25]. In the last decade, many experiments have been performed on the nanocomposite (used graphene oxide, nanotube, carbon quantum or other nanomaterial) membranes [26,27,28,29,30,31], which may be the harbinger of the next generation of membranes [16].

The accurate determination of the osmotic pressure is central to analyzing these processes, for key system parameters, such as water flux, power density, and membrane structural parameter, depend on osmotic pressure. The non-ideality of sodium chloride solutions complicates the evaluation of osmotic pressure. As discussed by Mistry and Lienhard [32,33], the rational activity coefficient, osmotic coefficient and osmotic pressure of NaCl and mixed electrolyte solutions can deviate significantly from those of an ideal solution (the rational activity coefficient varies from 0.67–1.2 for a molality of 0–6 mol_NaCl_/kg_water_). Nonetheless, the literature often uses the ideal van ’t Hoff linear equation (π = *iCRT*), valid only for dilute solutions [34], to predict osmotic pressure in aqueous solutions [2,8,12,13,16,24]. We compare the van ’t Hoff prediction for osmotic pressure to that of the OLI software (OLI Stream Analyzer 2.0 software [35]), which gives the real osmotic pressure values as a function of the solute concentration [36,37], for aqueous NaCl solutions. The error in the van ’t Hoff prediction, in some cases over 10–15% depending on the draw solution concentration, then propagates to other system parameters (water flux, power density, membrane structural parameter [38,39,40]) derived from the predicted osmotic pressure. These parameters are necessary to evaluate the separation efficiency of asymmetric [41] or thin film composite membranes [2,42,43] in PRO and FO. In particular, the membrane structural parameter *S* crucially determines membrane performance [8,10,12,38,40]. In addition, many papers have studied the mass transport in hollow fiber membrane modules [44,45,46,47], but transport models are not the topic of this paper.

Several reviews have also been conducted on models and methods for determining the structural parameter, *S* [48,49,50]. Cath et al. [2] worked on a method to standardize membrane characterization in forward osmosis. Lee et al. [38,51] showed that membrane surface porosity can significantly affect water and solute flux values, which serve as the basis for structural parameter calculations. Manickam and McCutcheon [40] discuss how the values of the predicted structural parameter can deviate by one to two orders of magnitude from those obtained through direct measurements [52]. The soft properties of the membrane layer prevent accurate direct measurements of this parameter [40]. Thus, mass transport models using fitted parameters based on measured water flux data [2,8,10,11,24,38,40,46,53,54] are used to predict *S* and are well accepted in the literature [12,24,46,54,55,56,57].

These models require accurate values of transport parameters, including external mass transfer coefficients, the bulk diffusion coefficient, and/or water (*A*) and salt (*B*) permeabilities. Loeb et al. [10] developed basic expressions for determining the structural parameter in PRO and FO. These expressions do not account for external concentration polarization (ECP) layers. Achilli et al. [1] extended this model to account for ECP. Yip et al. [42] later combined ECP with internal concentration polarization (ICP) to calculate solute transport. Nagy [54] and Bui et al. [12] accounted for all four transport layers (active layer, ICP, ECP on both sides of the membrane) in their transport models. Although mass transport models accounting for all layers exist, authors typically use Loeb’s model [10] for predicting the structural parameter [2,8,37,42,58,59].

Experimentally, two sets of commercially available membranes were investigated, namely, a thin-film nanocomposite membrane (NanoH_2_O), with inorganic nanoparticles incorporated in the selective layer [60], and the asymmetric Porifera membrane [61,62]. The main objective of these experiments was twofold. Firstly, the experiments were conducted to determine the main characteristic properties (*A*, *B*, *R*) of the membranes. And secondly, these characteristic properties were used to calculate values of the structural parameters to show the effect of the osmotic pressure’s prediction on their values, for comparison with data from the literature [37,40].

This paper aims to determine whether the discrepancy between van ’t Hoff’s approach and OLI software predictions in osmotic pressure leads to inaccurate prediction of the water flux, power density, membrane structural parameter, and separation efficiency for NaCl solutions or whether these errors are negligible in system analyses of PRO and FO. For the conditions under which the van ’t Hoff approach yields misleading values of system parameters, such as water flux and power density, the value of the structural parameter will be determined. The impact of external mass transfer coefficients on the predicted value of the structural parameter will also be investigated, using mass transport models that account for all transport layers [12,24,46,54,55,56,57].

## 2. Materials and Methods

This section is an overview of the applied membranes and the experimental, theoretical methods.

### 2.1. Materials

#### 2.1.1. Membranes

Two different flat-sheet membrane layers were chosen for investigation: the Porifera (PFO) membrane, which is commonly used for FO and PRO applications (manufactured by Porifera Inc., Hayward, CA, USA); and the NanoH_2_O membrane, which is commonly used for Reverse Osmosis (RO) (NanoH_2_O Inc. Torrance, CA, USA, recently LG NanoH_2_O Inc., Torrance, CA, USA). The NanoH_2_O membrane has a thin film nanocomposite polyamide active layer containing inorganic nanoparticles. The active layer has a thickness of 500–900 nm with a polysulfone support layer. This two-layer membrane is supported by a non-woven fabric layer to strengthen the mechanical stability. The thickness of the NanoH_2_O flat-sheet membrane is around 200 μm.

Porifera (PFO) is an asymmetric, two-layer flat-sheet membrane, consisting of a Nomex^®^ polymer, which is durable and heat resistant. The polymer contains aromatic groups in its molecular structure [61]; the overall thickness of the membrane was measured to be between 70–90 μm.

#### 2.1.2. *A*, *B* and *R* Values of Investigated Membranes

Permeabilities can be determined experimentally for reverse osmosis (RO) or, e.g., through simultaneously fitting *A*, *B*, and *S* [8]. In this study, the values of *A* [63] and *B* [64], measured by conventional methods, are considered as intrinsic membrane parameters. Cross-flow experiments on RO using deionized water DI water water at velocities ranging from 0.20–0.25 m/s and pressures ranging from 3–9 bar were conducted to determine the water permeability *A,* solute permeability *B*, and percentage of solute rejection *R* (Table 1). Porifera and NanoH_2_O membranes were investigated using the PRO operating mode (draw solution faces the selective layer) and FO operating mode (draw solution faces the support layer). However, the results for Porifera membranes in FO mode are not shown in this study due to the low obtained values of *R*. The real cause of this behavior needs further investigations; it is not the aim of this paper. Three trials tests were carried out for both membranes. The solute rejection is determined from the concentration difference between the bulk feed and permeate salt concentration: *R* = 1 − *C_p_/C_b_*, while the *B* value is obtained by *B* = *J_w_*(1 − *R*)/*R*.

### 2.2. Methods

#### 2.2.1. Membrane Characterization

**Figure 1:**
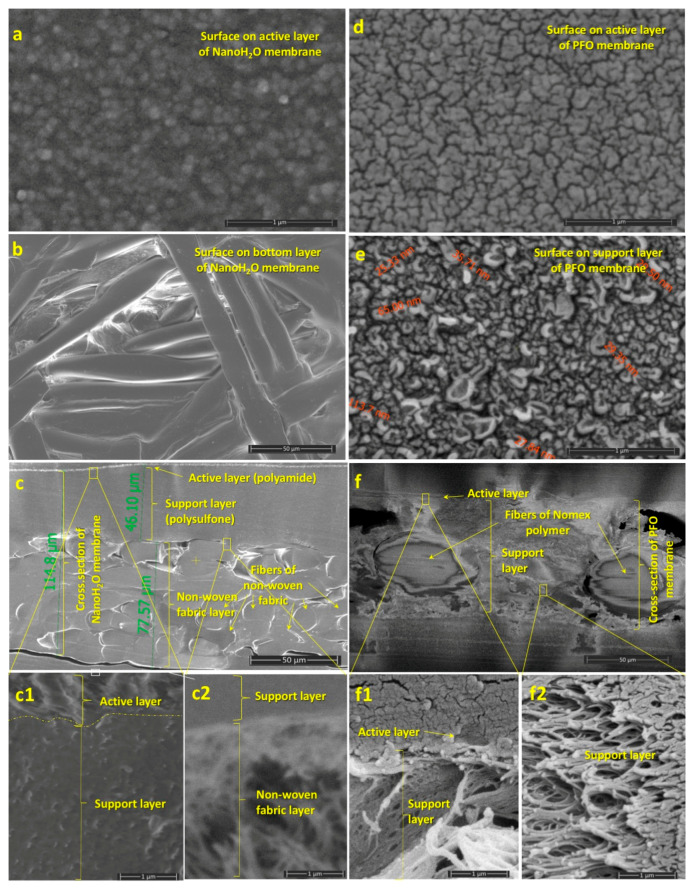
SEM images of the surface and cross-section structure of two membranes used in experiments: QuantumFlux (NanoH_2_O) membrane (**a**–**c**) and PFO (Porifera) membrane (**d**–**f**). (**a**) Surface structure of the active layer of NanoH_2_O membrane; (**b**) surface structure of the support, or non-woven fabric, layer of NanoH_2_O membrane with observable circular fibers (15–25 μm thick); (**c**) the cross-section of NanoH_2_O membrane contains a thin (500–900 nm) active layer and a thick (70–80 μm) porous support layer, which can result in unique separation features; (**d**) the active surface of PFO membrane; (**e**) the surface of the support layer of PFO membrane; (**f**) the cross-section of PFO membrane contains a very thin active layer (**f1**) and porous sponge-like support layer (**f2**). PFO membrane fibers of Nomex polymer (50 μm thick) are also well detectable.

Microstructure and pore size distributions of PFO and NanoH_2_O membranes were tested by an FEI/ThermoFisher Apreo S scanning electron microscope (ThermoFisher Scientific, Waltham, MA, USA). Observations made by SEM were carried out in a low vacuum with an accelerating voltage of 2.0 and 5.0 kV, depending on the electron-beam sensitivity of the samples. In order to acquire the best analytical results for the microstructure, the samples were mounted in a methyl methacrylate casting resin (Dentacryl Technicky, SpofaDental Inc., Jicin, Czeh Republic) and cut with a PowerTome-PCZ (RMC) type ultramicrotom. Since the resin is non-conductive by nature, the resin mounted samples conductive for SEM analysis, a JEOL IB-29510VET-type carbon evaporator (JEOL, USA Inc., Ltd., Peabody, MA, USA) was used, preparing a thin electron transparent layer (approximately 15–25 nm) by carbon (Figure 1a–c,c1,e,f,f1,f2) and gold (Figure 1d) onto the sample surface.

#### 2.2.2. Determination of the Pure Water and the Solute (NaCl) Permeability Coefficients

The pure water permeability, *A*, was determined by measuring the water permeate rate in the RO mode, applying hydraulic transmembrane pressure differences, ranging. The applied hydraulic pressures ranged from 2 to 10 bar, at a cross-flow velocity of 0.1–0.25 m/s, at 22 °C. In addition, the pure water permeance was also measured by a dead-end filtration method by pressurizing the liquid with an inert gas. The intrinsic salt rejection and the salt permeability coefficient were measured using 2 g/L NaCl solution, in the hydraulic pressure ranges between 2 and 9 bar. Values of *A*, *B* and *R* were calculated using expressions given in the literature [41,46].

#### 2.2.3. Determination of the Osmotic Pressure

The osmotic pressure of the NaCl solution was predicted using two different methods. Firstly, the van ’t Hoff approach (π = *iRTC*, where *R* is the universal gas constant, *T* is the absolute temperature, and *i* is the van ’t Hoff factor) was used to calculate the osmotic pressure. In addition, the OLI software (OLI Stream Analyzer 2.0 software) [35] was also employed for a relative comparison. The approach uses the following curve-fit expression for the osmotic pressure, π = 5.94028*C*^2^ + 37.4521*C*, based on the values produced by the OLI software. Here, *C* is the actual solute concentration in mol/L, and π is the osmotic pressure in bar, as given by the OLI software when *C* > 0.6 mol/L. For values of concentration below 0.6 M, values were linearly interpolated at intervals of 0.01 g/g using tabulated OLI data shown in Ref. [35].

#### 2.2.4. Evaluation of the Osmotic Water Flux and Solute Flux

The water flux and the reverse salt fluxes on both the NanoH_2_O and Porifera membranes were determined in parallel using devices from Beroplan GmbH (St. Inberg, Germany) and a custom-made, cross-flow FO set-up. The membrane areas used across the Beroplan GmbH and the in-house setup were 50 and 90 cm^2^ membrane surfaces, respectively. This membrane module is comprised of two geometrically similar channels on both sides of the membrane. Both liquid phases were recirculated separately through 2L-2L reservoirs, in closed loops. In certain cases (measuring the pure water permeability and the salt permeability by the RO method), a high-pressure peristaltic pump was used to circulate the draw solution. The change in the solutions’ weight was monitored over time. The concentration change in the liquid phases was measured using a conductivity meter. Experiments were performed by orienting the membrane in both PRO (active membrane layer facing the draw solution) and FO (active layer facing the feed solution) modes. The cross-flow velocity was kept between 0.2 and 0.25 m/s at 22 °C, without the presence of a hydraulic pressure difference. The draw solution concentration was chosen to be between 0.25 and 1.5 M NaCl, while DI water was used as the feed solution. The conductivity of both the solutions was monitored to determine the reverse salt flux across both membranes. The external mass transfer coefficients were predicted using data from [12].

#### 2.2.5. Prediction of the Structural Parameter

Known theoretical expressions, i.e., Equations (13) and (15), for cases of *k_d_*→∞ and *k_f_*→∞, and the presented ones, Equations (16) and (17), were applied for prediction of the values of the structural parameter, *S.* These were conducted using the measured water flux data under the previously specified operating conditions. There has been no direct, acceptable measurement’s method for the determination of the structural parameter until now. The main aim of this paper is to compare the values of the structural parameter obtained by the two different methods of the osmotic pressure’s calculations and their alignment with experiments. The in-house computer program written by us for these calculations uses QuickBASIC software. The high accuracy of this computer program (with accuracy of 16 decimals) was applied for the prediction. The stepwise variations of the *J_w_* values were compared to those measured until the relative difference between them was less than 0.01%.

## 3. Theoretical Development

The power density in PRO is determined by calculating the differences between the osmotic pressure and hydraulic pressure on both sides of the asymmetric membrane. The concentrations and osmotic pressures on both sides of the membrane active layer can be expressed explicitly. The individual concentration values of the solute on either side of the membrane selective layer were defined by Nagy [46,54] and Nagy et al. [36,55] for both PRO and FO operation modes. This enables the user to determine the osmotic pressure separately on both sides of the active layer, and thus the osmotic pressure difference. The concentration difference obtained using these individual interface values should be the same as that given by the conventional expressions (Equation (3) or (6) below). Knowledge of the individual osmotic pressures can be advantageous when the linear relationship between the ratio of concentration and the osmotic pressure (e.g., as assumed by Lee et al. [59]) is not valid.

**Figure A1:**
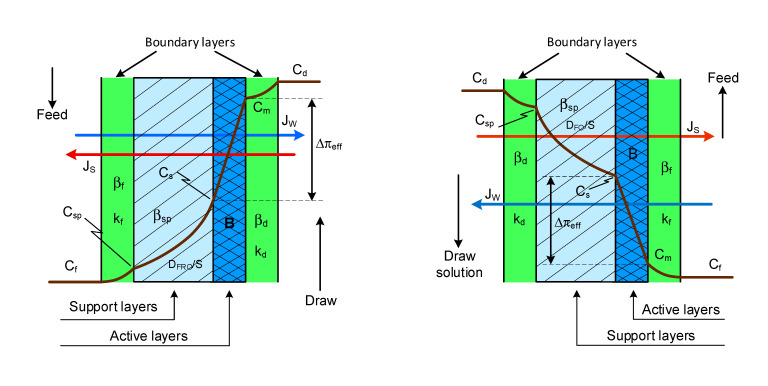
Illustration of the concentration distribution and nomenclature of parameters for PRO (left-hand side figure) and FO (figure on the right-hand side) processes, including external mass transfer resistances. *C_m_* and *C_s_* correspond to the interface concentration of the active layer facing the boundary layer and facing the porous support layer, respectively. Δπ_eff_ is the effective osmotic pressure difference on the active layer.

The notation established for the interface concentrations and other nomenclature used during the course of this study is illustrated in Appendix A (Figure A1): *C_s_* denotes the concentration between the selective and support layers in both operation modes (PRO and FO), while *C_m_* denotes the interface concentration between the selective layer and draw side boundary layer (in the case of PRO) or feed side boundary layer (in the case of FO).

The interface concentrations are given by the following expressions ([46], pp. 519–521).

Case A: Pressure-retarded osmosis (PRO).

(1) $$C_{m}=\frac{C_{d}\left\{1+\frac{B}{J_{w}}\left(e^{J_{w}\left(S/D+1/k_{f}\right)}-1\right)\right\}e^{-J_{w}/k_{d}}+C_{f}\frac{B}{J_{w}}\left(1-e^{-J_{w}/k_{d}}\right)e^{J_{w}\left(S/D+1/k_{f}\right)}}{1+\frac{B}{J_{w}}\left(e^{J_{w}\left(S/D+1/k_{f}\right)}-e^{-J_{w}/k_{d}}\right)}$$

(2) $$C_{s}=\frac{C_{d}\left\{\frac{B}{J_{w}}\left(e^{J_{w}\left(S/D+1/k_{f}\right)}-1\right)e^{-J_{w}/k_{d}}\right\}+C_{f}\left\{1+\frac{B}{J_{w}}\left(1-e^{-J_{w}/k_{d}}\right)\right\}e^{J_{w}\left(S/D+1/k_{f}\right)}}{1+\frac{B}{J_{w}}\left(e^{J_{w}\left(S/D+1/k_{f}\right)}-e^{-J_{w}/k_{d}}\right)}$$

The concentration difference between the two sides of the selective layer is determined by subtracting Equation (2) from Equation (1). This provides the following mathematical expression for the pressure-retarded osmosis case:

(3) $$\Delta C_{m}\equiv C_{m}-C_{s}=\frac{\left(C_{d}e^{-J_{w}/k_{d}}-C_{f}e^{J_{w}\left(1/k_{f}+S/D\right)}\right)}{1+\frac{B}{J_{w}}\left(e^{J_{w}\left(1/k_{f}+S/D\right)}-e^{-J_{w}/k_{d}}\right)}$$

Case B: forward osmosis (FO).

The interfacial concentrations are provided below ([46], pp. 447–456, [55]):

(4) $$C_{s}=\frac{C_{d}\left\{1+\frac{B}{J_{w}}\left(e^{J_{w}/k_{f}}-1\right)\right\}e^{-J_{w}\left(S/D+1/k_{d}\right)}+C_{f}\frac{B}{J_{w}}\left(1-e^{-J_{w}\left(S/D+1/k_{d}\right)}\right)e^{J_{w}/k_{f}}}{1+\frac{B}{J_{w}}\left(e^{J_{w}/k_{f}}-e^{-J_{w}\left(S/D+1/k_{d}\right)}\right)}$$

And

(5) $$C_{m}=\frac{C_{d}\frac{B}{J_{w}}\left(e^{J_{w}/k_{f}}-1\right)e^{-J_{w}\left(S/D+1/k_{d}\right)}+C_{f}\left\{1+\frac{B}{J_{w}}\left(1-e^{-J_{w}\left(S/D+1/k_{d}\right)}\right)\right\}e^{J_{w}/k_{f}}}{1+\frac{B}{J_{w}}\left(e^{J_{w}/k_{f}}-e^{-J_{w}\left(S/D+1/k_{d}\right)}\right)}$$

The concentration difference between the two sides of the selective layer is similar, calculated by taking the difference between Equation (4) and Equation (5):

(6) $$\Delta C_{m}\equiv C_{s}-C_{m}=\frac{\left(C_{d}e^{-J_{w}\left(1/k_{d}+S/D\right)}-C_{f}e^{J_{w}\left(1/k_{f}\right)}\right)}{1+\frac{B}{J_{w}}\left(e^{J_{w}\left(1/k_{f}\right)}-e^{-J_{w}\left(1/k_{d}+S/D\right)}\right)}$$

### 3.1. Prediction of the Osmotic Pressure Difference, *Δ*π_m_

The osmotic pressure difference can be determined using the interface concentrations at each side of the membrane selective layer for either PRO and FO. Accordingly, the PRO osmotic pressure difference is:

(7) $$\Delta \pi =\pi_{m}\left(C_{m}\right)-\pi_{s}\left(C_{s}\right)$$

The OLI software determines the osmotic pressure of the NaCl aqueous solution as a function of the water activity in conjunction with an activity coefficient model:

(8) $$\pi \left(C\right)=-\frac{RT}{\bar{v}}lna_{w}$$

where $\bar{v}$ is the partial molal volume of water, and *a_w_* is the activity of water. The activity can be rewritten as the product of the fugacity coefficient of the solvent and its mole fraction. Knowing the concentrations on both sides of the active layer makes it possible to determine the osmotic pressure difference using Equation (7), using the concentrations given by Equations (1) and (2).

Using the van ’t Hoff approach, namely π = *iRT C*, where *R* is the universal gas constant, *T* is the absolute temperature, and *i* is the van ’t Hoff factor (number of ions). Then, by multiplying Equation (1) by *iRT*, the osmotic pressure at the draw side of the skin layer can be predicted via the van ’t Hoff approach.

(9) $$\pi_{m}\left(C_{m}\right)=\frac{\pi_{d}\left\{1+\frac{B}{J_{w}}\left(e^{J_{w}\left(S/D+1/k_{f}\right)}-1\right)\right\}e^{-J_{w}/k_{d}}+\pi_{f}\frac{B}{J_{w}}\left(1-e^{-J_{w}/k_{d}}\right)e^{J_{w}\left(S/D+1/k_{f}\right)}}{1+\frac{B}{J_{w}}\left(e^{J_{w}\left(S/D+1/k_{f}\right)}-e^{-J_{w}/k_{d}}\right)}$$

Similarly, for the other side of the active layer, the linearized osmotic pressure takes the following form:

(10) $$\pi_{s}\left(C_{s}\right)=\frac{\pi_{d}\left\{\frac{B}{J_{w}}\left(e^{J_{w}\left(S/D+1/k_{f}\right)}-1\right)e^{-J_{w}/k_{d}}\right\}+\pi_{f}\left\{1+\frac{B}{J_{w}}\left(1-e^{-J_{w}/k_{d}}\right)\right\}e^{J_{w}\left(S/D+1/k_{f}\right)}}{1+\frac{B}{J_{w}}\left(e^{J_{w}\left(S/D+1/k_{f}\right)}-e^{-J_{w}/k_{d}}\right)}$$

**Figure A2:**
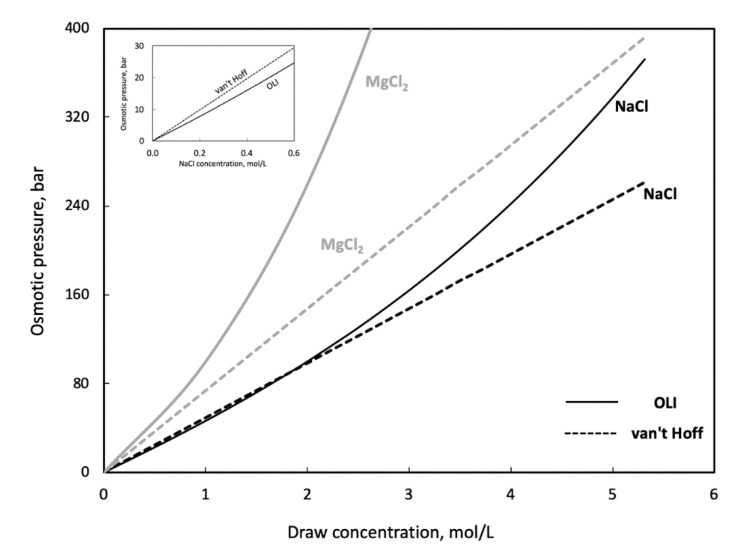
Osmotic pressure, predicted by the van ’t Hoff linear approach and the OLI Streamline Analyzer software, as a function of the solute concentration for NaCl and MgCl_2_. The vertical axis corresponds to the osmotic pressure.

The osmotic pressure of NaCl and MgCl_2_ solutions as a function of concentration [4,35,46] is illustrated in Figure A2. Here, osmotic pressure has been plotted using both the van ’t Hoff approach and by the OLI Stream Analyzer. The individual data points represent the measured data. The continuous line is a fitted equation of the following form: π = 5.94028*C*^2^ + 37.4521*C*, where *C* is the actual solute concentration in mol/L, and π is the osmotic pressure in bar, as given by the OLI software. At lower solute concentrations (C ≤ 0.6 M), the tabular data were linearly interpolated at intervals of 0.01 g/g. This method provides additional resolution and accuracy in determining the osmotic pressure. Knowing the individual osmotic pressures, π(*C_m_*) and π(*C_s_*), the PRO osmotic pressure difference can be calculated by solving for the difference between both terms, as seen by Equation (7). For FO mode, the terms on the right-hand side of Equation (7) are reversed.

For the van ’t Hoff approach, the PRO-mode osmotic pressure difference can be calculated by taking the product of Equation (3), the universal gas constant, the van ’t Hoff factor, and the temperature (seen in Equation (11).

(11) $$\Delta \pi \equiv \pi_{m}-\pi_{s}=\frac{\left(\pi_{d}e^{-J_{w}/k_{d}}-\pi_{f}e^{J_{w}\left(1/k_{f}+S/D\right)}\right)}{1+\frac{B}{J_{w}}\left(e^{J_{w}\left(1/k_{f}+S/D\right)}-e^{-J_{w}/k_{d}}\right)}$$

The osmotic pressure difference can be similarly calculated for the FO operating mode, by applying the van ’t Hoff linear expression to Equation (6). The result is:

(12) $$\Delta \pi \equiv \pi_{s}-\pi_{m}=\frac{\left(\pi_{d}e^{-J_{w}\left(1/k_{d}+S/D\right)}-\pi_{f}e^{J_{w}\left(1/k_{f}\right)}\right)}{1+\frac{B}{J_{w}}\left(e^{J_{w}\left(1/k_{f}\right)}-e^{-J_{w}\left(1/k_{d}+S/D\right)}\right)}$$

The water flux can be obtained by calculating the product of the osmotic pressure difference and the water permeability, *A* [$J_{w}=A\left(\Delta \pi -\Delta P\right)$] The flux takes the following equation form for FO mode, when there is no hydraulic pressure difference: $J_{w}=A\Delta \pi$. Similarly, the solute flux can be determined using the concentration differences at the membrane surface and the salt permeability, *B*. The solute flux equation follows the same format, $J_{s}=B\Delta C_{m}$, and is consistent for both operating modes (PRO and FO).

The solution procedure requires an initial guess for the water flux to compute the concentrations on both sides of the membrane. The guess is updated by recalculating the flux through the product of the osmotic pressure and the water permeability. This process is iterative and stopped when the difference between the previously iterated flux value and the updated one is less than 0.01%.

### 3.2. Prediction of the Membrane Structural Parameter

Determining intrinsic membrane properties such as water (*A*) and salt permeability (*B*) is generally conducted through reverse osmosis (RO) experiments that independently vary hydraulic pressure, as illustrated in the literature [8,56,57,63,64]. The value of *A* is obtained from the gradient of water flux vs. the hydraulic pressure difference. *B* is obtained by means of the rejection value of the solute component and the water flux ([46], p. 525). The structural parameter is often predicted by measured water flux data in a system run in both the pressure-retarded osmosis (PRO) and/or FO operating mode, neglecting the effect of the external mass transfer resistances [8,12,37,40]. Loeb et al. developed an expression for the prediction of the membrane structural parameter [10]. Accordingly, its value can be obtained for both the PRO and FO modes.

For PRO mode (the draw solution faces the membrane skin layer), in case of *k_d_→∞* and *k_f_→∞* [2,40,45,52,58,65,66,67,68,69] (*J_w_*_,∞_ represents the water flux while *S*_∞_ means the value of structural parameter, both obtained without external solute transfer resistances):

(13) $$S=\frac{D}{J_{w}}ln\frac{B-J_{w}+A\pi_{d}}{B+A\pi_{f}}$$

Or the water flux can be expressed from Equation (13) as:

(14) $$J_{w}=\frac{D}{S}ln\frac{B-J_{w}+A\pi_{d}}{B+A\pi_{f}}.$$

At a given *S* value, the water flux can be predicted by this relatively simple expression. The water flux predicted by Equation (14) should give the same results as those obtained using Equation (11) in the limiting case, i.e., *k_d_→∞* and *k_f_→∞*.

For FO mode (the draw solution faces the membrane support layer), for the case of *k_d_→∞* and *k_f_→∞* [4,10,58,70,71]:

(15) $$S=\frac{D}{J_{w}}ln\frac{B+A\pi_{d}}{B+J_{w}+A\pi_{f}}$$

Equations (13) and (15) define the value of the structural parameter as a function of more easily determinable intrinsic parameters. These include *A* and *B* as well as the water flux rate, *J_w_* without external transfer resistances, and the diffusion coefficient in the bulk fluid phase, *D*. This procedure does not involve any external mass transport resistances. Since Loeb’s paper [10,58] was published, transport models have regularly taken into account one of the external boundary layer resistances, i.e., the one facing the active membrane layer [1,8,42,72]. More recently developed models also take into account both of the external fluid phase resistances [12,46,54,55,56]. These most recent transport expressions involve the effect of all four transport layers (see Figure A1). These resistances can be introduced into Loeb et al. equations resulting in the expressions in Equations (16) and (17) for the case of *k_d_* ≠ ∞ and *k_f_* ≠ ∞.

For the PRO mode, applying Equation (7) or Equation (8) with $J_{w}=A(\Delta \pi -\Delta P)$:

(16) $$S=\frac{D}{J_{w}}ln\frac{B-J_{w}e^{J_{w}/k_{d}}+A\pi_{d}}{\left(B+A\pi_{f}\right)e^{J_{w}\left(1/k_{f}+1/k_{d}\right)}}$$

For the FO mode, applying, e.g., Equation (12) and $J_{W}=A\Delta \pi$:

(17) $$S=\frac{D}{J_{w}}ln\frac{B+A\pi_{d}}{\left(B+J_{w}e^{-J_{w}/k_{f}}+A\pi_{f}\right)e^{J_{w}\left(1/k_{f}+1/k_{d}\right)}}$$

Equations (16) and (17) clearly show that the external resistance adjacent to the support layer, *k_f_,* can also have a significant impact in determining the structural parameter. The degree to which this effect can influence the predicted value of the structural parameter is similar in the extent to the external resistance of the active layer. Thus, neglecting this external resistance might also cause significant errors in prediction of the actual values of *S*, as discussed in Section 3.2.

It can be noted that relatively accurate predictions of the external mass transfer coefficients, *k_d_*, *k_f_*, as well as *A*, *B*, and *D*, are necessary to obtain a precise value for the structural parameter. Therefore, if these relevant parameters are calculated to a high degree of accuracy, the correctness of the applied membrane mass transfer model used for both the PRO and FO processes should provide accurate and real values for the *S* structural parameter. Considering the measured values of *A* and *B* as intrinsic parameters, their determined values can be regarded as relatively accurate [35]. As mentioned previously, the external mass transfer resistances are also important for determination of the salt permeability [2,12,68,71]. It can, however, be stated that the predicted *k_f_* and *k_d_* values are usually practically well-determined ones, and, therefore, their predicted values should not introduce too much error into the values of the membrane structural parameter. The value of the diffusion coefficient in the pores of the support layer remains uncertain. It is generally acceptable to assume that the diffusion coefficient value within the support layer is equal to its bulk fluid diffusion coefficient value.

Conversely, the measured values of *A* and *B* should also be independent of the RO process used for their determinations [35]. Thus, the previously experimentally determined *A* and *B* values can really be regarded as accurate. Moreover, a reliable prediction of the external mass transfer coefficients is still required for correct determination of the *S* value. This can be done using the measured water flux data. Taking into account the effect of the external mass transfer resistance, during determination of the salt permeability [2,12,68,71] is also critical for accuracy. The predicted *k_f_* and *k_d_* values are already well determined. Consequently, their measured/predicted values do not lead to large errors in the values of the membrane structural parameter.

## 4. Results and Discussion

### 4.1. Characterization of the NanoH_2_O and the Porifera (PFO) Membranes

Figure 1 presents selected micrographs of the NanoH_2_O (Figure 1a–c,c1,c2) and Porifera (Figure 1d–f,f1,f2) membrane layers. The NanoH_2_O membrane consists of three layers with the following thicknesses: a polyamide active layer (500–900 nm), a microporous polysulfone support layer (about 50 μm), and a structural support, or non-woven fabric, layer comprised of cylindrical fibers (15–25 μm) [73]. Figure 1a,b show the surface of the top (active) and bottom (non-woven fabric layer) of the NanoH_2_O membrane, respectively. Given the limitations of the electron microscopy we employed, the nano-scale pore size distribution on the active layer surface is not visible. Figure 1c represents the complete cross-section of this membrane, with magnified images shown in Figure 1c1,c2. The polyamide active layer contains approximately 50–100 nm thick cavities [74], reflecting its greater porosity relative to the polysulfone support layer. However, these cavities are covered and connected by a nonporous polyamide layer of 20 nm thickness, which is permeable only to water molecules [75]. The non-woven fabric layer provides the strong mechanical stability of the membrane. The three membranes layers, particularly the non-woven fabric layer, may significantly hinder solute transport relative to water transport, i.e., increase solute transport resistance against osmotic pressure difference [60]. This phenomenon is further investigated in Section 4.2.

Similarly, the PFO membrane consists of a thin and compact active layer (500–900 nm) and a thick porous support layer (70–80 μm). The active layer surface does not contain detectable pores (<20–50 nm) at micrometric or sub-micrometric scales (Figure 1d), unlike the support layer surface that contains microscopic pores (30–100 nm) (Figure 1e). Figure 1f shows the cross-section structure of the PFO membrane. Cylindrical fibers of Nomex polymer (about 50 μm thick) are detectable in the support layer. The support layer does not have the usual capillary microstructure. The wall of the capillary-like structure contains numerous holes, giving it a sponge-like character (Figure 1f2).

### 4.2. Osmotic Performance of Membranes

**Figure 2:**
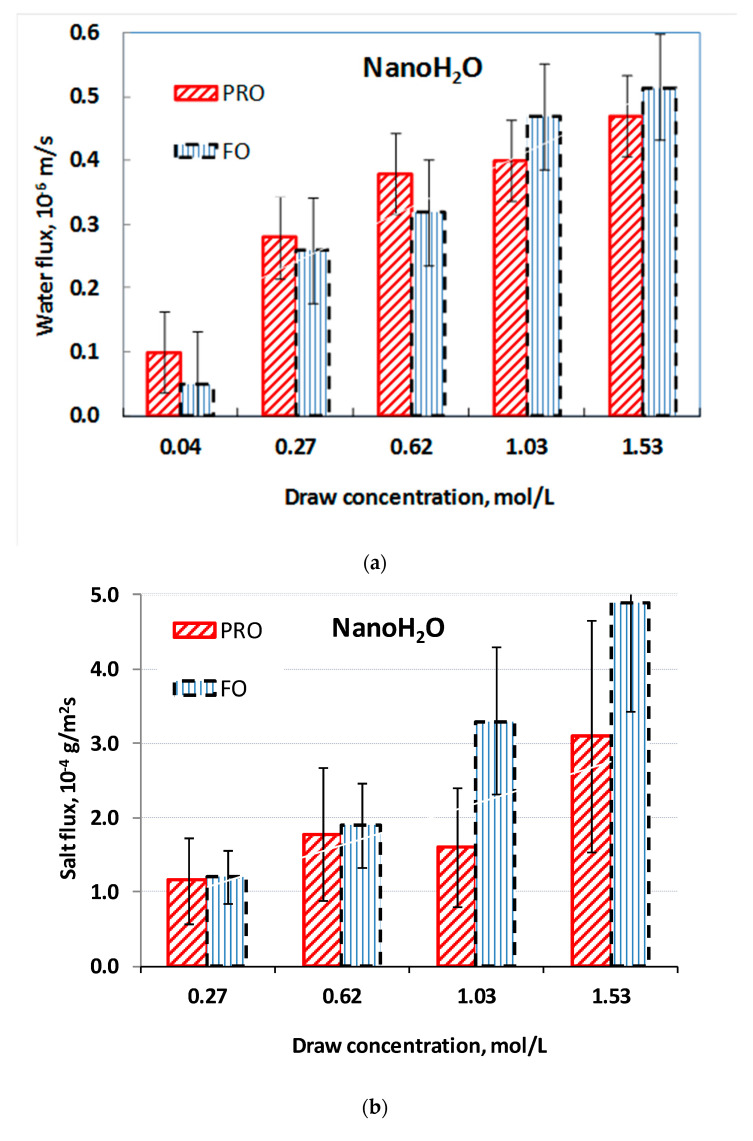
(**a**) Osmotic water fluxes of the NanoH_2_O membrane in pressure-retarded osmosis (PRO) and forward osmosis (FO) modes. Experiments were performed at 22 °C and 0.20–0.25 m/s cross-flow velocity with DI water at the feed side and without a hydraulic pressure difference (1 × 10^−6^ m/s = 3.6 Lmh). (**b**) Salt fluxes of the NanoH_2_O membrane in PRO and FO modes. Experiments were performed at 22 °C and 0.20–0.25 m/s cross-flow velocity with DI water at the feed side and without a hydraulic pressure difference.

**Figure 3:**
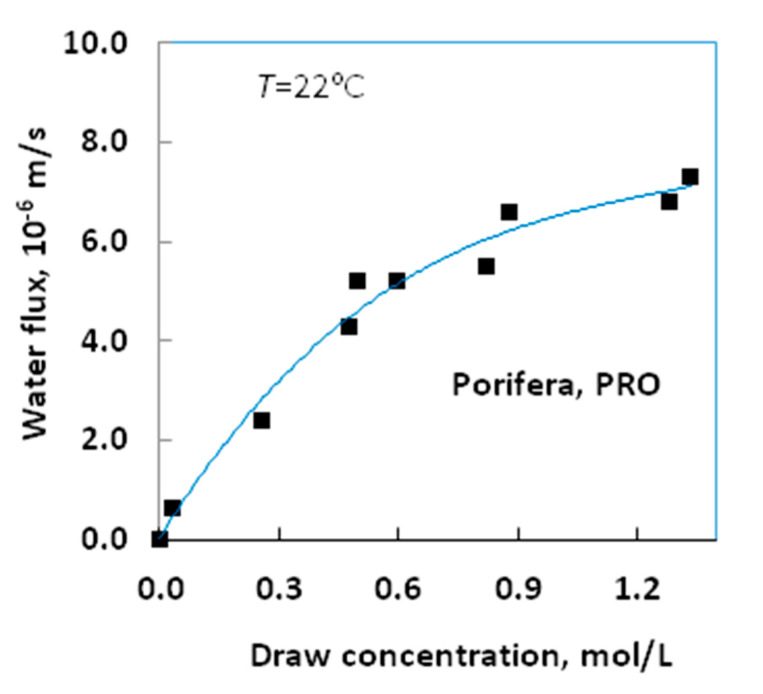
Osmotic water flux of the Porifera membrane as a function of the draw NaCl concentration in PRO mode. Experiments were performed at 22 °C and at 0.20–0.25 m/s cross-flow velocity with DI water at the feed side and without a hydraulic pressure difference (the continuous line is a fitted curve to the measured points).

Figure 2a shows the water flux of the NanoH_2_O membrane measured in PRO and FO modes, with five NaCl dilutions acting as the draw solutions and DI water acting as the feed solution. The water flux in FO mode is higher at larger draw concentrations than PRO mode. This finding is unexpected, because draw solute concentration, in PRO mode, typically generates larger water flux in asymmetric membranes. However, the NanoH_2_O membrane is an atypical asymmetric membrane due to the nanosized particles on its selective layer. The water flux data are approximately one-order of magnitude lower than those of the cellulose triacetate membrane from [40] (see Section 4.4.2, it discussed later) and the Porifera membrane in PRO mode (Figure 3). This low water flux, despite the NanoH_2_O membrane’s permeability, may be caused by the fabric layer as well as the rather dense support layer hindering water transfer, thereby reducing the effect of osmotic pressure relative to the hydraulic pressure difference ([62] reached a similar conclusion). The salt fluxes of the NanoH_2_O membrane in both operating modes are shown in Figure 2b. The salt flux and the uncertainty of the measured flux is somewhat higher in FO mode compared to PRO mode.

Figure 3 demonstrates the osmotic water flux as a function of the draw solute concentration, with DI water on the feed side, for the Porifera membrane. Our values match those obtained by Motsa and Bamba [62]. Variations in measured water flux even at similar draw concentrations support the high error of approximately ±20%, similar to those given by [40]. The substantially higher Porifera water fluxes relative to those of the NanoH_2_O membrane suggest that the Porifera membrane is more suitable for use in the PRO process.

### 4.3. Theoretical Analyses of the Membrane Performance with Typical Parameter Values

When evaluating experimental data, to what extent does the choice of osmotic pressure prediction method affect the predicted values of the structural parameter, *S*? In this section, we compare results based on two prediction methods, the van ’t Hoff approach and the OLI stream analyzer, using NaCl as the solute compound.

The water flux and power density are plotted and briefly discussed as a function of draw solute concentration, hydraulic pressure difference, membrane structural parameter, water permeability, and solute permeability. Other parameter values, listed in Table 2, are held constant. The membrane parameters chosen here reflect the performance of today’s more typical CTA membranes, which differ substantially from the Porifera and NanoH_2_O measurements. The effect of the external mass transfer coefficients on the predicted values of the structural parameter is also shown in some figures, using data of [36], and discussed in Section 4.4.2 of the main text, using measured water flux data from [37,40], demonstrating the significance of their effect on the evaluation of the measured data.

The solution pair applied here is seawater as the draw solution (*C_d_* = 0.6 M, i.e., ~35 g/L) and river water as the feed solution (*C_f_* = 0.015 M, i.e., ~0.9 g/L). The effect of values *C_d_*, *k_d_* and *k_f_* are briefly discussed for PRO and FO systems. Accurate characterization of the solute bulk diffusion coefficient is important because it proportionally affects the predicted value of the membrane structural parameter (Equations (13)–(17)). The value of the fluid diffusion coefficient varies by less than 5% across the range of relevant solute concentrations, namely, between 1.323 and 1.383 × 10^−9^ m^2^/s at 20 °C and *C* = 0–1.5 M, as given by [12] or 1.510–1.556 × 10^−9^ m^2^/s at *C* = 0–60 g/L according to [1,8,76]. The solute diffusivity was chosen to be 1.367 × 10^−9^ m^2^/s (Table 2), according to the prediction by Tow and Lienhard [77].

In the next Section 4.3.1, Section 4.3.2 and Section 4.3.3, the data of Table 2 are used for the prediction of the water flux and the effect of the membrane or operating parameters as a function of one of the varied parameters.

#### 4.3.1. Effect of Osmotic Pressure and Transport Parameters on Water Flux and Power Density

The osmotic pressure is predicted by using both the van ’t Hoff linear approach and the OLI System Analyzer [35], which gives real values of the osmotic pressure as a function of the solute concentration. The osmotic pressure of NaCl solution as a function of concentration according to both prediction methods is shown in Figure A2 (see Appendix B). The water flux and power density are compared, using both osmotic pressure prediction methods, as a function of the draw solute concentration, hydraulic pressure difference, membrane structural parameter, solute permeability, and water permeability.

##### Effect of Draw Concentration on Power Density

In cases of most solute components, the osmotic pressure increases super-linearly with solute concentration [4]. This function can strongly depend on the solute component and the number of ions. For example, the osmotic pressure of the MgCl_2_ is significantly higher than that of sodium chloride [4], because the van ’t Hoff factor I, which is related to the number of solute ions, is equal to three for MgCl_2_ and two for sodium chloride. Figure A2 shows the predicted osmotic pressure using both the van ’t Hoff equation and the OLI software in cases of both NaCl and MgCl_2_ solute compounds. The left-hand ordinate shows the osmotic pressure of the NaCl solution, while the right-hand ordinate the MgCl_2_ solution. Values obtained by the van ’t Hoff approach fall on the same curve in the two cases because the ratio of their values is equal to 3/2. This figure clearly illustrates the high difference between the osmotic pressure values (more detailed discussion is given in the Appendix B). Accordingly, significantly different results can be expected of the two prediction methods when evaluating the water flux or the power density. This deviation will also affect the calculated value of the structural parameter when based upon water flux measurements.

**Figure 4:**
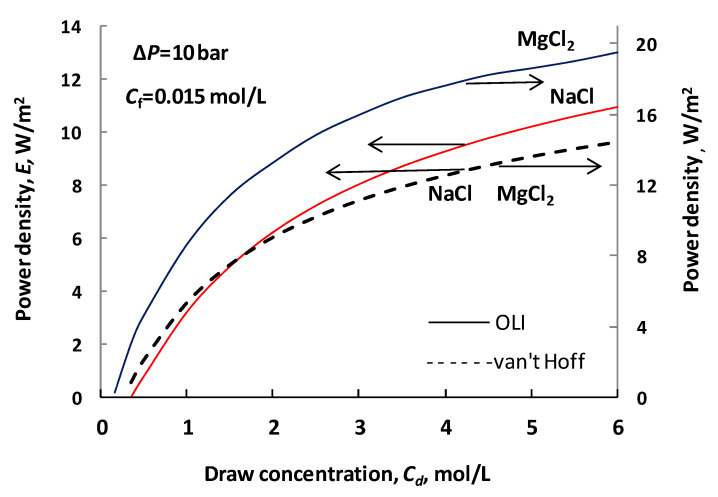
Predicted power density/water flux as a function of the solute concentration for PRO and FO systems comparing the linear approach and the OLI Stream Analyzer’s data [35] for the osmotic pressure. (Other parameters are listed in Table 2).

Figure 4 illustrates the change in power density (note that according to *E* = *J_w_* Δ*P*, 1 W/m^2^ power density is obtained by *J_w_* = 10 × 10^−6^ m/s water flux in case of Δ*P* = 10 bar), as a function of the draw concentration (values of other parameters are listed in Table 2; values of *A*, *B*, the bulk diffusion coefficient were chosen according to [36]; chosen values of *S* and the external mass transfer coefficients, *k_d_*, *k_f_*, are typical). Let us first look at the curves obtained for NaCl solutions, in both PRO mode (draw solution facing the skin layer) and FO mode (draw solution facing the support layer). At a solute concentration greater than about 1.9 M, the predicted power density is higher when using the OLI Stream Analyzer. The difference in power densities obtained by the two prediction modes gradually increases and reaches 14% at *C_d_* = 6 M. On the other hand, the van ’t Hoff approach predicts higher power densities at low concentrations with the seawater-river water pair (Figure A2 illustrates the osmotic pressure in the concentration range less than 0.6 M, in the upper part of this figure, on the left-hand side). In that concentration range (up to about 1.9 M draw concentration) the linear approach overestimates the osmotic pressure, which is shown in the inset of Figure A2.

Additionally, the power density is also plotted in this figure in the case of MgCl_2_ draw solution in PRO mode. The relative difference between the power densities of these two solutes decreases from 350% to 35%, in the concentration range of 0.6 and 3 M (see curves obtained by OLI software in Figure 1). Looking at the osmotic pressures, e.g., at *C_d_* = 3 M, the differences between the van ’t Hoff and OLI models are about 15 and 275 bar for NaCl and MgCl_2_, respectively (see Figure A2). Against the huge difference in osmotic pressure, e.g., at *C_d_* = 3 M, the difference in power densities of the two components (~35%) is rather moderate. This is the consequence of the solute transport resistances in the membrane support layer and in the external boundary layers.

**Figure 5:**
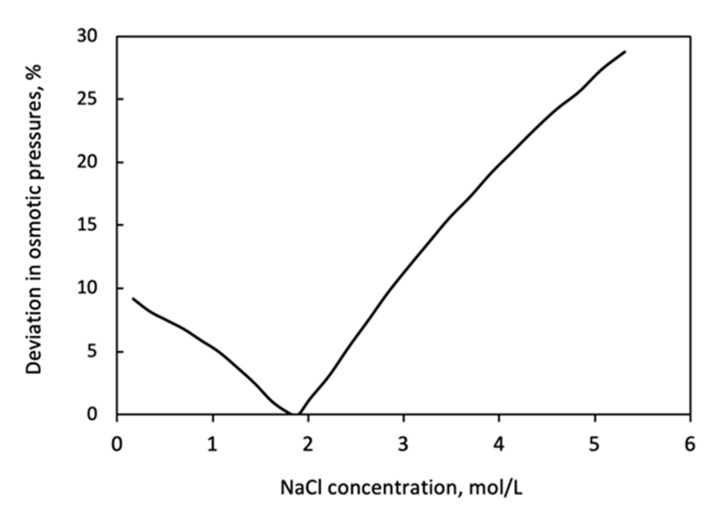
Change of the relative error in percent ($\left|\pi_{lin}-\left.\pi_{OLI}\right|\right./\pi_{x}$, where π*_x_* indicates the higher pressure in each case) as a function of the solute concentration, applying for prediction of the π value by the van ’t Hoff linear approach and the OLI software.

The relative values of deviation are illustrated by Figure 5, as a function of the solute concentration. (The difference is related to the higher osmotic or hydraulic pressure values.) This figure well illustrates the error in osmotic pressure caused by applying the van ’t Hoff linear equation, comparing its data to those of OLI software ones. If one assumes that the acceptable error should be falling below 5%, then this is valid in a concentration range of 1 to 2.4 M. The intersection point, where the error is equal to unity, is at about 1.9 M. The deviation can reach even the 30% above 2.4 M solute concentration, where its value increases with the concentration. Figure 5 clearly illustrates the concentration range, in that the linear approach can be acceptable, perhaps between 1 and 2.4 M NaCl concentration range as draw solution. However, it should be noted that the real effect of this deviation on the value of the mass transport parameter can vary, so we consider each parameter in the following sections.

##### Effect of Hydraulic Pressure Difference on Water Flux and Power Density

**Figure 6:**
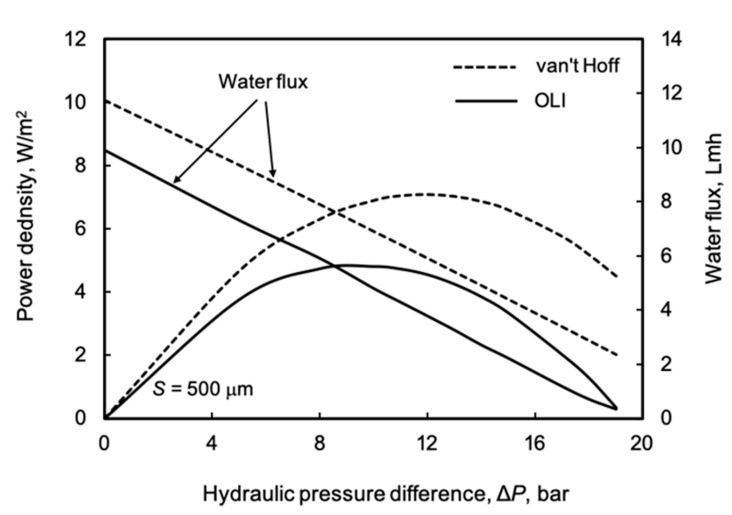
Power density/water flux as a function of the hydraulic pressure difference applying the van ’t Hoff approach and the OLI software for prediction of the osmotic pressure (other parameters are listed in Table 2).

The effect of the hydraulic pressure difference on water flux and power density is illustrated by the commonly used river water and seawater pair, applying again the data listed in Table 2 (Figure 6). The shape of these curves is well known from the literature [1]. It is well known that there is a linear relationship between the water flux, *J_w_*, and the hydraulic pressure difference, Δ*P: J_w_* = *A*(Δπ − Δ*P*). There exists an optimal hydraulic pressure difference that provides the maximum power density. In our case, it is important to see the differences in power density between the two prediction modes! It varies, increasing strongly with the increase in power density. Let us show the difference, e.g., at Δ*P* = 12 bar, where the power density has approximately its maximum value under the van ’t Hoff approach. The harvestable energy values are 7.1 and 4.9 W/m^2^, as obtained by the linear approach and OLI software, respectively. Thus, the deviation between the two prediction methods reaches 30%, which is significant. At higher osmotic pressures, the difference further increases. This clearly illustrates that the application of the linear or van ’t Hoff approach to describe the solute transport and membrane performance can lead to significant errors (more than 100% at lower Δ*P* values, not shown here) in the evaluation of transport in the river water-seawater pair.

##### Effect of the Membrane Structural Parameter, *S* on Water Flux

**Figure 7:**
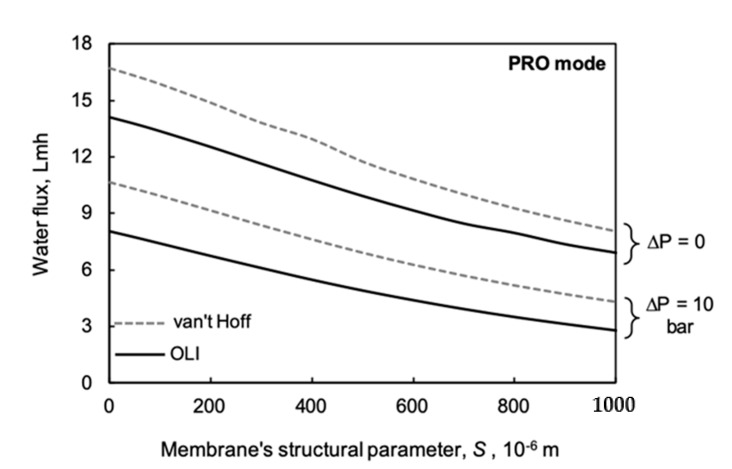
Water flux as a function of the membrane structural parameter at two different values of the hydraulic pressure (Other parameters are listed in Table 2) (1 Lmh = 0.2778 × 10^−6^ m/s).

The membrane structural parameter is crucially important to membrane performance. It is generally the greatest mass transport resistance of the four different mass transfer layers, illustrated in Figure A1. Lowering its value is one of the most important tasks for the industrial producers of membranes. The effect of the *S* value on water flux is plotted in Figure 7 using the parameter values listed in Table 2. Two different Δ*P* values are chosen, i.e., Δ*P* = 0 and 10 bar. Generally, the deviation obtained by the two prediction methods increases with increasing hydraulic pressure difference, which is in harmony with data given in Figure 6. On the other hand, the data obtained by OLI software are remarkably lower than those obtained by the linear approach. Let us emphasize water flux data, e.g., at *S* = 500 μm and Δ*P* = 10, as examples. The difference in predicted water flux is about 30%. At increasing values of the membrane structural parameter, the relative error in predicted water flux increases. These results show that the usage of the van ’t Hoff linear approach can give larger errors in water flux/energy density as the structural parameter increases, even for low values of the draw concentrations, such as those paired with seawater.

##### Effect of the Water Permeability, *A*, on Power Density

**Figure 8:**
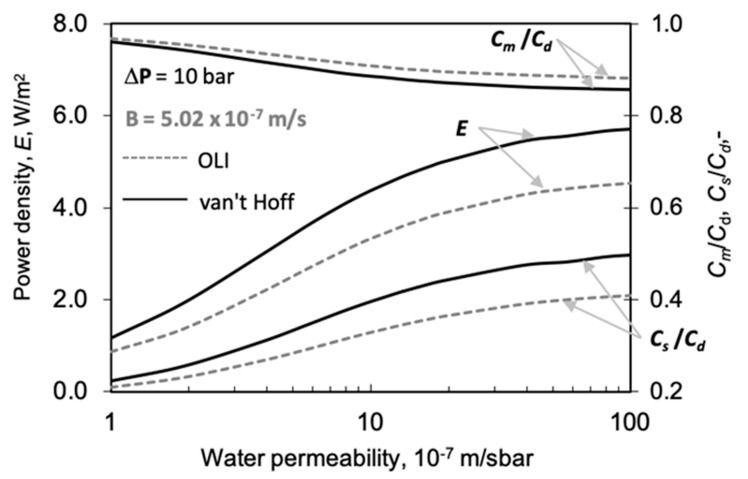
Power density and concentration of the membrane active layer as a function of the water permeability (constant parameters are given in Table 2).

In this subsection and the next subsection, we analyze the effect of water permeability and solute permeability on power density. Together, these two key parameters (so called ‘intrinsic membrane parameters’) decisively determine membrane performance. The value of the water permeability determines the power density of a PRO membrane process. Figure 8 illustrates the strong effect of *A* on the power density (note that according to *E* = *J_w_* Δ*P*, 1 W/m^2^ power density is obtained by *J_w_* = 1 × 10^−6^ m/s water flux, at Δ*P* = 10 bar; accordingly, the value of *E* in Figure 5 corresponds to that water flux). Other transport parameters are kept constant as listed in Table 2. It can be clearly seen in Figure 8 that the difference in power density, as obtained by the two prediction methods, gradually increases with water permeability. On the other hand, the relative error does not increase that much, e.g., at *A* = 1.9 × 10^−7^ m/s-bar and 10 × 10^−7^ m/s-bar, the relative difference of the value of *E* were obtained to be 29% and 23%, respectively, due to the stronger increase in the absolute value in water fluxes, which values are 1.36 × 10^-6^ and 4.4 × 10^−6^ m/s, at these *A* values.

We also show how the active layers’ solute concentrations, *C_m_* and *C_s_*, vary as a function of the water permeability. The role of the internal and external mass transfer resistances gradually increases with the increase in water flux (these are related to values of *C_m_/C_d_* and partly to value of *C_s_/C_d_*), which then can moderate the increase in the water flux or power density. This is a consequence of the water and solute transport occurring in opposite directions. Due to this, the increased water flux increases the overall resistance of the boundary layers [46,55]. Though the value of Δ*C_m_* (=*C_m_* − *C_s_*) gradually decreases, the power density increases due to the more rapidly increasing value of the water permeability or water flux. On the other hand, the two prediction methods give remarkably different concentration values. It is also worth noting that the value of *E* is higher for the van ’t Hoff approach, though the relative value of Δ*C_m_* will be lower with increasing value of *A* (dotted lines of *C_m_/C_d_* and *C_s_/C_d_* in FO mode), but its absolute value gradually increases, due to the larger increase in the difference in their absolute values (not shown here).

##### Effect of the Solute Permeability, *B*, on Power Density

**Figure 9:**
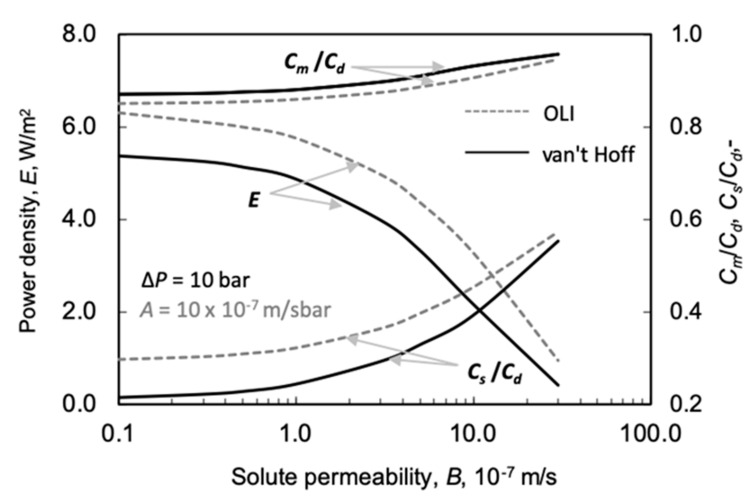
Power density and concentration of the membrane active layer as a function of the solute permeability (Remained parameter are given in Table 2).

The solute permeability, *B*, also has a huge effect on the power density (Figure 9). A relatively high value was chosen for the water permeability here: *A* = 10 × 10^−7^ m/s·bar. The power density decreases strongly, with an accelerated tendency, as a function of the value of *B*. The difference between the two prediction methods for the calculation of the osmotic pressure here is also rather essential. The relative difference in values of *E*, obtained by the two prediction methods gradually increases with solute permeability, though the absolute difference between them somewhat lowers due to their lowering absolute values. At solute permeability values of *B* = 5 × 10^−7^ and 10 × 10^−7^ m/s, the differences in power density obtained by the prediction methods are 24% and 33%, respectively. Increasing *B* value lowers the concentration difference across the active membrane layer and thus decreases the water flux and power density, as illustrated in Figure 9.

Summarizing the results discussed in the above subsections, it can be stated that the van ’t Hoff equation and the OLI Stream Analyzer software give remarkably different results using the seawater and river water pair. Figure A2 excellently illustrates the difference in the predicted osmotic pressure in the lower concentration range. Accordingly, for the evaluation of the measured results in PRO systems, the OLI software is recommended if one wants to get more realistic membrane performance data on the energy-producing processes.

#### 4.3.2. Membrane Structural Parameter, *S*

It seems there is no direct measuring method for determination of the value of the membrane structural parameter. Its real value depends on the thickness of the membrane, on the membrane porosity, ε, and on the membrane tortuosity, τ:*S* = δτ/ε.

In this subsection, measured water flux, *J_w_*_,∞_, [37,40] is applied for the prediction of the value of the structural parameter, i.e., the values of *S/S*_∞_.

**Figure 10:**
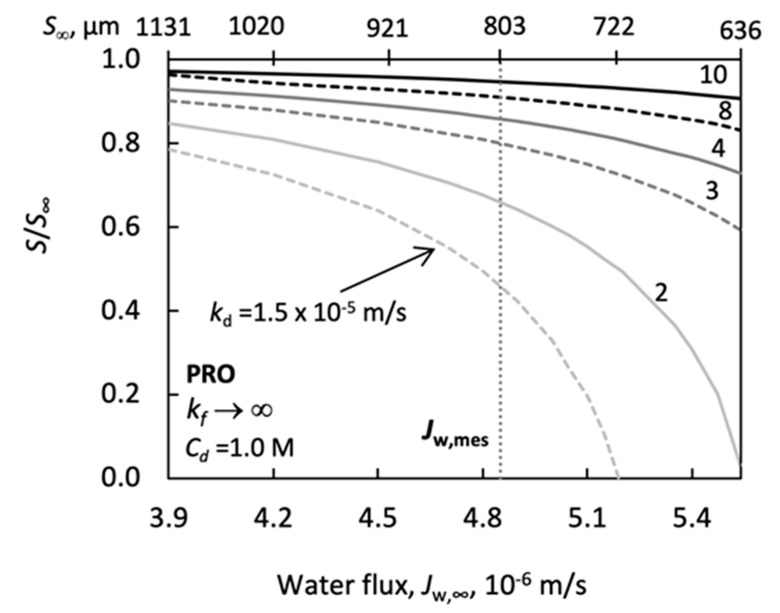
Change of the *S/S_∞_* values as a function of the water flux in a PRO system, taking into account their predicted error of the measured data (±20%), given by Manickam and McCutcheon [40]. Parameter is the external mass transfer coefficient in the boundary layer facing the active membrane layer, at *C_d_* = 1.0 M, and *k_f_→*∞ (*A* = 1.71 × 10^−7^ m/s·bar; *B* = 1.94 × 10^−7^ m/s; *C_f_* = 0; *S* = 803 × 10^−6^ m; Δ*P* = 0; *D* = 1.5 × 10^−9^ m^2^/s). On the upper horizontal axis, the calculated value of the corresponding structural parameter is given for *k_d_*→∞, *k_f_*→∞.

**Figure 11:**
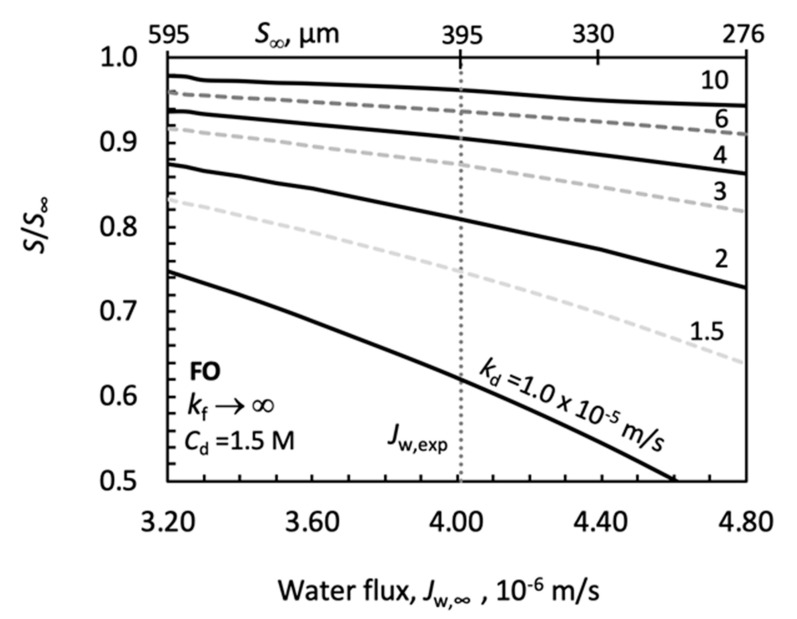
Change of the *S/S_∞_* values as a function of the water flux in the FO system, taking into account ±20% error of the measured data, at *C_d_* = 1.5 M, *k_f_*→∞ applied data of Manickam and McCutcheon [40] (other parameters are given in caption of Figure 10). On the upper horizontal axis, the calculated value of the corresponding structural parameter, *S*_∞_, is given for *k_d_→*∞, *k_f_*→∞.

**Figure 12:**
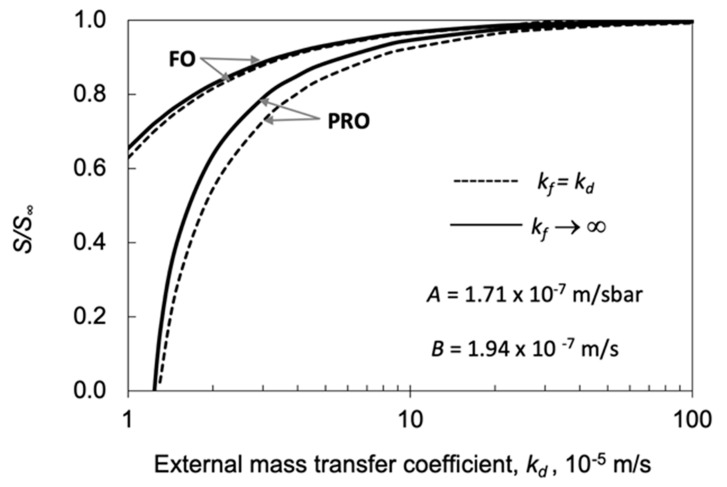
Value of *S/S_∞_* as a function of the draw side mass transfer coefficient with both operating modes, i.e., PRO and FO; *C_d_* = 1.0 M; *k_f_→*∞; for PRO: *J_w_,*_∞_ = 4.92 × 10^−6^ m/s; *S*_∞_ = 803 μm; for FO: *J*_∞_ = 3.0 × 10^−6^ m/s; *S*_∞_ = 395 μm; other parameters as they are given in caption of Figure 10.

**Figure 13:**
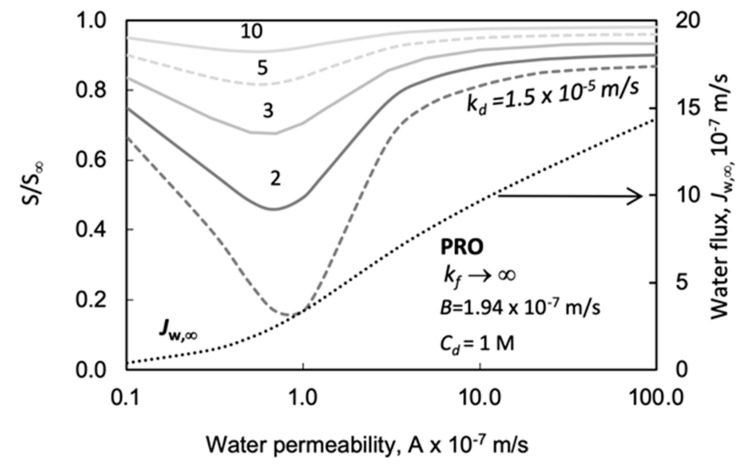
Effect of the water permeability on the value of *S/S*_∞_ and on the water flux, *J_w_*_,∞_, as a function of draw side mass transfer coefficient, in a PRO system; *C_d_* = 1.0 M; *S_∞_* = 803 μm; π*_d_* = 43.3 bar; *k_f_→*∞; other parameters as they are given in caption of Table 5.

**Figure 14:**
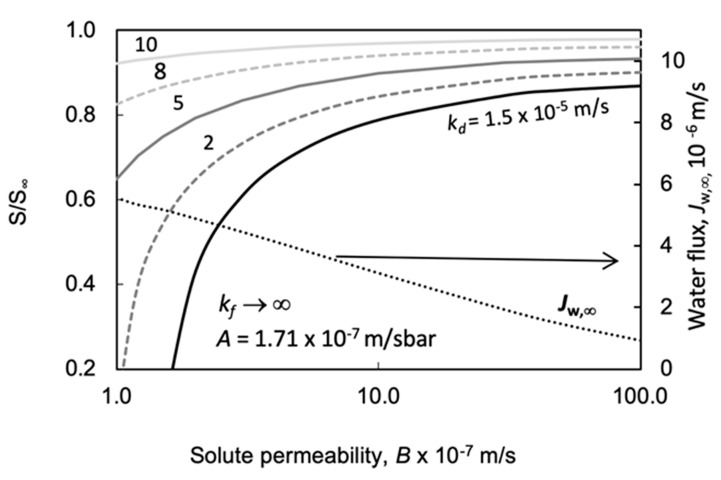
Effect of the solute permeability on the value of *S/S*_∞_ and on the water flux, *J_w_*_,∞_, as a function of draw side mass transfer coefficient, in a PRO system; *C_d_* = 1.0 M; *S_∞_* = 803 μm; *k_f_*→∞; other parameters as in Table 5.

The effect of *k_d_* was calculated by measured values of water flux, for both of PRO (Figure 10) and FO (Figure 11) systems, taking into account the error of the measured data, given by Manickam and McCutcheon [40] (it is about ±20%), as well as data of Tang et al. [37]. The effect of the low concentration side mass transfer resistance (1/*k_f_*→0) was neglected here for the sake of simplification. Additionally, the effect of the external mass transfer resistances on the *S/S*_∞_ was plotted in Figure 12. The effect of changing *A* and *B* are also illustrated (Figure 13 and Figure 14), for constant values of *S*. Then, the measured water flux data were used for prediction of the values of *S* and discussed both without and with the draw side mass transfer coefficient. Their results are listed in Table 3, Table 4, Table 5 and Table 6.

##### Effect of the Draw Side Mass Transfer Coefficient in PRO Mode

Let us develop a suitable expression for the prediction of the *S/S*_∞_ values, first for a PRO system. Equations (13) and (16) clearly show that the fluid phase mass transfer coefficients can strongly affect the value of the membrane structural parameter. The ratio of these equations can define the real effect of these parameters. This ratio can then easily be obtained, e.g., for a PRO system, as (it is worth noting that the measured water flux, *J_w_*_,_*_exp_*, is replaced in the following expression; its value is assumed to be equal to *J_w_*_,∞_ without external mass transfer resistance, i.e., *J_w_*_,_*_exp_* ≡ *J_w_*_,∞_):

(18) $$\frac{S}{S_{\infty }}=\frac{ln\left\{\left(B-J_{w,exp}e^{J_{w,exp}/k_{d}}+\pi_{d}A\right)/\left(B+\pi_{f}A\right)\right\}-J_{w,exp}\left(1/k_{d}+1/k_{f}\right)}{ln\left\{\left(B-J_{w,exp}+A\pi_{d}\right)/\left(B+A\pi_{f}\right)\right\}}$$

where *S* in the numerator of Equation (18) means the value of the structural parameter of the support membrane layer in the presence of the external mass transfer coefficients, while *S_∞_* is that without taking into account the external mass transfer resistances, i.e., *k_d_*→∞*,k_f_*→∞, i.e., values of these mass transfer coefficients are enough high, that the value of, e.g., exp(1/*k_f_*) ≅ 1 and that of *k_d_*, as well. Equation (18) is a simple, explicit expression for prediction of the effect on the membrane structural parameter in presence of external mass transfer resistance. The value of *J_w_*_,_*_exp_* simply represents the measured water flux, independent of whether or not external mass transfer resistances are present. In order to get water flux without external mass transfer resistance, such operating conditions should be provided where the assumption that *k_d_*→∞, *k_f_*→∞ is fulfilled. During our recalculation of data, the value of the structural parameter was predicted by the trial-error method (see Section 4.4). Value of *S*/*S_∞_* expressed by Equation (18) is equal to unity, when *k_d_*→∞, *k_f_*→∞, and its value gradually decreases with the increase of any value of the external mass transfer coefficients, though to different degrees. Equation (18) clearly shows that both external mass transfer resistances can affect the predicted values of *S*, although not in the same manner. The water flux also varies with the change of the *k_d_* and/or *k_f_* values, when other parameters are constant.

Figure 10 illustrates the effect of the draw side mass transfer coefficient on the value of *S/S*_∞_ as a function of the water flux. The water flux was changed, taking into account its measured value (*J_w_* = 4.9 × 10^−6^ m/s, dotted line in Figure 10; see also Table 4, at *C_d_* = 1.0 M) and its error, in the range of ± 20%, i.e., between 3.8 × 10^−6^ and 5.5 × 10^−6^ m/s. As can be seen, the value of the draw side external mass transfer coefficient depends strongly on the water flux. In principle, any of the water flux data, used in this calculation, could have been the measured one. It is obvious that the value of the structural parameter varies with the change of the water flux. Values of *S*_∞_ are listed on the upper part of this figure. Let us look at values of *S/S*_∞_ at *J_w_*_,_*_exp_* (≡ *J_w_*_,∞_; dotted, vertical line). It varies between 0.95 and 0.42 when *k_d_* values are decreased from 10 × 10^−5^ m/s down to 1.5 × 10^−5^ m/s. The measured values of the *k_d_* can change between about (2 and 4) × 10^−5^ m/s [12,77] or even higher [1], at cross-flow velocities of 20–30 cm/s. These *S/S*_∞_ data underscore the requirement that the measured water flux data be very carefully evaluated regarding the draw side and feed side mass transfer resistances. More precise determination of the external mass transfer coefficients is needed and one should avoid neglecting the external resistances.

##### Effect of the Draw Side Mass Transfer Coefficient in FO Mode

The effect of the external mass transfer coefficients under PRO mode is essentially different from that obtained in the FO system. This behavior is caused by the fact that the solute concentrations on the two sides of the active layer are much lower in FO mode due to the lower interface concentrations caused by the resistance of the membrane support layer. Accordingly, the concentration difference in the active layer can also differ from those obtained in the case of PRO systems ([46], pp. 450–453). Against that, analysis of the FO operation mode can also be useful. Let us express the value of *S/S_∞_* for this separation mode, using Equations (15) and (17) for FO mode:

(19) $$\frac{S}{S_{\infty }}=\frac{ln\left\{\left(B+\pi_{d}A\right)/\left(B+J_{w,exp}e^{-J_{w,exp}/k_{f}}+\pi_{f}A\right)\right\}-J_{w,exp}\left(1/k_{d}+1/k_{f}\right)}{ln\left\{\left(B+A\pi_{d}\right)/\left(B+J_{w,exp}+A\pi_{f}\right)\right\}}$$

It is worth noting that the measured water flux is assumed to be equal to that obtained without external mass transfer resistance, i.e., *J_w_*_,_*_exp_* ≡ *J_w_*_,∞_. It is worth noting that *k_f_* represents the external mass transfer coefficient on the feed side, which faces the skin membrane layer in FO operation mode (Figure A1). *S/S_∞_* as a function of the draw side water flux, taking into account the about 20% error of the measured *J_w,∞_* value, is shown in Figure 8, considering the measured value published in [40], at *C_d_* = 1.5 M. Other data can be found in Table 2. Values of the structural parameter are also given in this figure. Different values of the draw side fluid mass transfer coefficient are plotted (value of *k_d_* is varied between 1.5 × 10^−5^ m/s up to 10 × 10^−5^ m/s), without transfer resistance on the feed side, *k_f_*→∞. The effect of the *k_d_* external mass transfer coefficient on the *S/S_∞_* value is significantly less than in PRO mode. Its effect may be neglected, at lower values of *k_d_*, only. On the other hand, the water flux affects the value of the structural parameter strongly, in the water flux range investigated.

##### Value of S/S_∞_ as a Function of Draw Side Mass Transfer Coefficient by Both of PRO and FO Operation

How *S/S*_∞_ varies as a function of the draw side mass transfer coefficient is shown in Figure 12, applying parameters’ data of Manickam and McCutcheon again [40], at *J_w_*_,_*_exp_* = 4.9 × 10^−6^ m/s, *C_d_* = 1.0 M, *S*_∞_ = 803 μm for PRO and *J_w_*_,__∞_ = 3.0 × 10^−^^6^ m/s; *C_d_* = 1.0 M; *S*_∞_ = 434 μm, for FO operation (see Table 5). Other constant parameter values are given in the caption of Figure 10. Generally, it can be stated that the effect of the external mass transfer coefficients can practically be neglected when values of these coefficients are larger than 10 × 10^−5^ m/s. Thus, e.g., the value of *S/S*_∞_ is equal to 0.92 for PRO and 0.97 for FO mode, at *k_d_* = 10 × 10^−4^ m/s, while there is no resistance on the permeate side. On the other hand, *S/S*_∞_ quickly decreases with the *k_d_* mass transfer rate between 1 × 10^−5^ m/s and 10 × 10^−5^ m/s (note here that the draw side mass transfer resistance faces the membrane support layer). It is worth noting that the value of the structural parameter without external resistance, S∞ and *J_w_*_,_*_exp_* were kept constant during the calculation; thus, in order to reach the measure water flux, the actual value of *S* should gradually be lowered. Zero value of *S/S*_∞_, at the given external mass transfer resistances, means that, to reach the value of *J_w_*_,_*_exp_*, the diffusion path of the membrane support layer, *S*, should be equal to zero. The sum of the external fluid layers and the active layer mass transfer resistances reaches the maximum value of the possible transport resistance. This figure also confirms that the effect of the draw side mass transfer resistance is much stronger; therefore, its effect should be taken into account in order to avoid essential error in the predicted value of the structural parameter. It can also be seen that the relative deviation of results between the two modes, with and without permeate side mass transfer resistance, continuously increases in PRO mode. Thus, the relative deviations between the two modes of osmotic pressure calculation, e.g., between *k_d_* = 2 × 10^−5^ m/s and *k_f_* = *k_d_* or *k_f_*→∞, as well as *k_d_* = 1.5 × 10^−5^ m/s and *k_f_* = *k_d_* or *k_f_*→∞, are 14% and 28%, respectively. The continuous lines represent data with external transfer resistance on both sides of the membrane (*k_d_* = *k_f_*), while the broken lines show data without external transfer resistance on the feed side.

Working under the usual operating conditions, i.e., with cross-flow velocity at about 0.2 and 0.5 m/s, the external mass transfer coefficients are mostly falling in the mass transfer coefficient range of 2 × 10^−5^ and about 8 × 10^−5^ m/s [1,12,77]. This should mean that the predicted value of the structural parameter will be significantly higher than its real values, which are obtained taking into account the external mass transfer resistances, at a given value of the water flux. The overestimation of the structural parameter then can lead to a faulty evaluation of membrane properties. Accordingly, the accurate prediction of values of the external mass transfer coefficients is crucially important for the correct evaluation of the membrane properties.

#### 4.3.3. The Effect of the External Mass Transfer Coefficient as a Function of A and B

Considering that both the water and solute permeability can significantly affect the water flux and, consequently, the effect of the external mass transfer resistances, as it is plotted in Figure 10, Figure 11 and Figure 12, the change of the water and the solute permeability were also studied and briefly discussed here. Figure 13 shows the variation of the *S/S*_∞_ value as a function of the water permeability, using the parameters in the caption of Figure 10 and at *C_d_* = 1.0 M; *S_me s_= S*_∞_ = 803 µm; *k_f_*→∞. The value of *S/S*_∞_ has a minimum as a function of *A*; the minimum occurs at larger *A* values as the external mass transfer coefficient decreases. The reason for this behavior in the value of *S/S*_∞_, in the *A* range of (0.1–1) × 10^−7^ m/s·bar, is that the value of *J_aw_/k_d_* increases by about an order of magnitude, and accordingly the increase in the numerator’s value is much less than that of the denominator in Equation (18), due to the increasing value of the second term of the right-hand side in this expression. On the other hand, with further increase in the value of *A* beyond 1 × 10^−7^ m/s·bar, the value of π*_d_A* in the numerator also gradually increases, pushing the value of *S/S*_∞_ up to unity. Values of *S/S*_∞_ vary significantly across the practical value range of the water permeability, i.e., between 1 × 10^−^^7^ m/s·bar and 8 × 10^−7^ m/s·bar. This figure clearly shows the importance of the *A* value in the real predicted values of the *S/S*_∞_ in the presence of an external mass transfer resistance. The water flux also strongly increases in the function of the *A* value (dotted line), as is expected.

How the solute permeability affects the predicted values of *S/S*_∞_ is illustrated by Figure 14 at *C_d_* = 1.0 M; *S*_∞_ = 803μm, *k_f_→*∞ (other constant parameter values as in the caption of Figure 10). A great variation in values of *S/S*∞ was obtained in Figure 14, especially at low values of the solute permeability. The external draw side mass transfer coefficient significantly affects the predicted value of *S/S_∞_*, in this value range of *B*. The water flux also changed as a function of the *B* value: its value lowers, close to linearly, with the increasing value of the solute permeability parameter. Comparing the change of *A* values in range between 1 × 10^−7^ m/s·bar and 100 × 10^−7^ m/s·bar (Figure 13), the change of the water flux is much higher than those in case of *B,* in the range of (1–100) × 10^−7^ m/s.

The data discussed in this and previous sections show that the change in value of all parameters can affect the measure of the effect of the external mass transfer resistance and on the predicted *S/S_∞_.* values. Accordingly, neglecting their effects without any critical considerations can cause an essential error in predicted *S* values. This should suggest that a prediction of the structural parameter should always carefully consider the possible effect of the external mass transfer rates, which depend mainly on the cross-flow velocity.

### 4.4. Evaluation of Measured Data Focusing on Values of the Membrane Structural Parameter

The value of the structural parameter cannot be directly measured. Thus, solute transport models are generally used to predict the *S* values. These models depend on measured water flux; membrane parameters *A*, *B*, and *D*; external mass transfer coefficients, *k_d_, k_f_*; and solute concentrations, or osmotic pressure π©. This section uses experimental water flux data measured by the authors and those published by Manickam and McCutcheon [40] and Tang et al. [37] in PRO and FO systems to investigate the effect of the commonly neglected external mass transfer coefficients on the structural parameter. We conclude that accounting for external mass transfer coefficients is necessary to accurately determine the structural parameter.

#### 4.4.1. Prediction of *S* Values Based on Measured Data Using NanoH_2_O and Porifera Membranes

The correct prediction of the structural parameter of the support layer is extremely important for the correct prediction of membrane performance, which gives essential information on the membrane’s quality. That is why not only experimental data are analyzed but detailed discussion of typical theoretical operating conditions will be shown. Accordingly, this paper offers a surveying evaluation of the membrane performance for both the PRO and FO systems. When evaluating experimental data, to what extent does the choice of osmotic pressure prediction method affect the predicted values of the structural parameter, *S*? In this section, we compare results based on two prediction methods, namely, the van ’t Hoff approach and the OLI stream analyzer, using NaCl as the solute.

Table 3 lists the predicted data of the structural parameter values of NanoH_2_O membrane using Equation (13) or Equation (16) for PRO and Equation (15) or Equation (17) for FO, in cases of *k_d_ = k_f_→*∞ and *k_d_ = k_f_* = 2 × 10^−5^ m/s, respectively, at the different draw concentrations. Values of *k_d_ = k_f_* = 2 × 10^−5^ m/s are regarded as actual ones for cross-flow velocity between 0.2 and 0.25 m/s, taking into account the measured data of [12]. It is remarkable the rather low values of the water flux compared to the typical commercial membranes, e.g., cellulose triacetate or thin-film composite membranes [12,42,75]. This low water flux data can be caused by the structure of the membrane. This may be caused partly by the compact character of the support layer and partly by the non-woven fabric layer, which can hinder the transport of the water molecules. This conclusion is in harmony with [62]. On the one hand, the FO system serves somewhat higher values of water flux at larger draw solute concentration. On the other hand, the rejection of the solute component (90.2%R; see Table 1) is remarkable in case of the FO operating mode.

As can be seen in Table 3, values of the structural parameter are unusually high, especially in the PRO system. On the other hand, values of *S* are significantly lower in the FO system than those in case of the PRO one. In the case of the PRO operating mode, these *S* values are surprisingly close two times higher than those obtained for PRO membranes, which are mostly below 1000 μm [37,40]. This large discrepancy is obviously caused by the compact structure of the support layer and also the non-woven fabric layer. Further detailed investigation is needed to clarify exactly the cause of this behavior.

The predicted values of the structural parameter for the Porifera membrane are listed in Table 4. All measured points, plotted in Figure 3, are taken into account. The average values do not involve the highest and the lowest measured points. Remarkably, the average data obtained by the van ’t Hoff approach and the OLI software give minor deviations. This difference is less than 4–5%. The average values of the structural parameter are also rather high, more than 1000 μm. Basically, these values are similar to those predicted in the literature [8,37,40].

#### 4.4.2. Prediction of *S* Values Based on PRO and FO Data in Manickam and McCutcheon

Manickam and McCutcheon [40] investigated the effect of the draw side concentration on the structural parameter for an FO system (Δ*P* = 0) operating in PRO (draw solution faces the active membrane layer) and FO (draw solution faces the membrane support layer) modes for two types of membranes. In our analysis, we consider cellulose triacetate membrane from Hydration Technology, USA, (HTI-CTA). Values of *A* and *B* were determined experimentally by the standard methodologies using RO. Several studies predict the *S* value of this membrane without accounting for external mass transfer resistances: Tiraferri et al. [8] predicted *S* = 481–541 µm, Kim et al. [9] *S* = 503–560 µm, Cath et al. [4] *S* = 450–560 µm, Achilli et al. [1] *S* = 675 µm, and Kim et al. [9] predicted *S* = 560–590 µm. The value of *S* for the HTI-CTA membrane, when accounting for external mass transfer resistances, is lower than that without resistance at a given measured value of *J_w_*. For example, Bui et al. [12] predicts *S* = 200–500 µm depending on the cross-flow velocity. The error in *S* increases as the external mass transfer resistances increase (see Figure 7, Figure 8, Figure 9, Figure 10 and Figure 11).

An iterative method using stepwise interpolation (0.01 g/g intervals) to determine *C* vs. π(*C*) at low concentrations (*C* < 0.6 M) and the expression π(*C*) = 5.94028 *C*^2^ + 37.4521 *C* for high concentrations (*C* > 0.6 M) are employed, for OLI prediction, to recalculate water flux without external mass transfer resistances, *J_w,∞_*, and with external mass transfer resistances, *J_w_*. Our objective is to ensure that our calculated water flux values are within 0.01% accuracy of the published water flux data. The *S* value is then fitted to these values of water flux using Equations (7) and (10) or (13). The *S/S_∞_* values are also calculated using Equation (18) for PRO and Equation (19) for FO (another approach is to calculate the *S*_∞_ value using Equation (13) for PRO or Equation (15) for FO in order to predict water flux from Equations (7) and (11) or (12)).

The experimental water flux data and predicted structural parameter data using the OLI software can be found in Table 5. For the sake of completeness, we also have predicted the values of structural parameter, applying the van ’t Hoff approach, as well and its error related to the OLI data. The deviation between results obtained by the two prediction methods is relatively high. Its value gradually decreases with an increase in the draw concentration, in harmony with the tendency of the osmotic pressure function, plotted in Figure 5. The predicted values of *S_∞_* (*k_d_ = k_f_→*∞) can deviate from those published by Manickam and McCutcheon, because *S* is sensitive to changes in water flux. The average value of *S* from Manickam and McCutcheon [40], 793 μm, matches the average value determined in this study, 794 μm, for PRO systems. The external resistance values, for *k_d_* = 5.0 × 10^−5^ and *k_d_*=2.0 × 10^−5^ m/s, are chosen based on those measured in the literature. (Bui et al. [12] finds *k_d_* = 1.74–1.84 × 10^−^^5^ and 2.0–2.1 × 10^−^^5^ m/s for 0–1.5 M NaCl concentrations and *k_f_* = 1.8–2.1 × 10^−^^5^ m/s for DI water with cross-flow velocities between 0.21 and 0.31 m/s. Manickam and McCutcheon [40] similarly measured *k_d_* = 2.0 × 10^−^^5^ m/s at 0.26 m/s cross-flow velocity. In contrast, other studies predicted or measured higher *k_d_* values, e.g., 4.8 × 10^−^^5^ m/s [77] or 8.5 × 10^−^^5^ m/s [1]).

For various water fluxes and draw concentrations, the ratio of our predicted *S* for these resistances relative to *S_∞_* ranges from 0.45 to 0.92 in PRO (Columns 5–6 in Table 5) and 0.77 to 0.93 in FO (Columns 5–6 in Table 5). These results illustrate that the external mass transfer coefficient can significantly impact the predicted values of the structural parameter, depending on the draw concentration. For example, at a water flux of 2.8 × 10^−6^ m/s and a draw concentration of 0.5 mol/L in PRO, the structural parameter, with *k_d_* = 2.0 × 10^−5^ and *k_d_* = 5.0 × 10^−5^ m/s, is 649 and 356 μm, respectively. These values are 82% and 45% of the structural parameter without external mass transfer resistance, which is 794 μm. Figure 10, Figure 11 and Figure 12 match the results in Table 5. The increase in the internal polarization layer’s resistance, i.e., an increase in *S*, may significantly reduce the effect of the external mass transfer coefficients. *S/S*_∞_ varies between 0.2 and 0.8 for *S* values of 636 to 1000 µm, respectively, at *k_d_* = *k_f_* = 2.0 × 10^−5^ m/s and for a decreasing *J_w_*_,∞_ (Figure 10). As *J_w_*_,∞_ increases, *S/S*_∞_ increases at approximately constant *S* (Table 5). Accordingly, the effect of the external polarization layer may depend on mass transport (*k_d_, k_f_, S*) and membrane (*A,B*) parameters (see Equations (3) and (7)). Consequently, neglecting this effect requires careful consideration.

#### 4.4.3. Prediction of *S* Values Based on PRO and FO Data in Tang et al.

Tang et al. [37] uses an FO system (Δ*P* = 0) operating in PRO and FO modes. They apply the van ’t Hoff linear approach for predicting osmotic pressure and mass transfer models without external mass transfer resistances for predicting structural parameter. However, Equations (14) and value of *J_aw_* expressed from Equation (15) should have been applied rather than Equations (7), (11) and (12) used in the literature, which are used by multiplying *A* for the calculation of the water fluxes, for the prediction of the water flux without external mass transfer resistances, *J_w,_*_∞_, at a given value of *S*_∞_. Knowing the value of *J_w_*_,∞_, the value of *S/S*_∞_ was predicted by Equations (18) or (19). We have re-evaluated the measured water flux based on osmotic pressures on both sides of the active membrane layer that were calculated using the OLI Stream Analyzer software [35]. Equations (16) and (17), accounting for the external mass transfer resistances, are used to predict the membrane structural parameters. Table 6 includes *S* values from Tang based on van ’t Hoff and our results based on OLI for various draw concentrations and measured water fluxes in PRO and FO. Results in Table 6 match with data shown in Figure 10 for PRO mode, and Figure 11; Figure 12 in FO mode. Our calculated *S* values without external resistances differ from those obtained by Tang et al., depending on draw solute concentration. This difference decreases with increasing draw concentration, in line with osmotic pressure deviations. For example, the *S* values largely vary from one another at the lower *C_d_* = 0.5 M, due to differences in osmotic pressure calculations using van ’t Hoff (24.3 bar) and OLI (20.2 bar). Beginning at approximately *C_d_* = 1.5 M, van ’t Hoff underestimates osmotic pressure, i.e., π(OLI) > π(van ’t Hoff). The effect of external mass transfer resistance on structural parameter similarly depends on *C_d_*, as *S/S*_∞_ increases with increasing draw concentration. Consistent with findings in Sec. 4.5.1, neglecting external resistance for external mass transfer coefficients larger than 4–5 × 10^−5^ m/s causes less error in the prediction of the membrane structural parameter.

We clearly show that the van ’t Hoff approach and the OLI software can yield different results, especially in the case of seawater–river water pairing, which has a low draw side concentration. This deviation strongly depends on the values of all transport parameters affecting solute and water transport rates.

## 5. Conclusions

The membrane structural parameter is a key indicator of membrane performance. This study investigates how the structural parameter calculation is affected by the osmotic pressure calculation and by the external mass transfer coefficients. Our analysis shows the importance of the careful application of these transport parameters in order to accurately predict membrane performance. We show that the van ’t Hoff approach does not agree with results based on the true osmotic pressure over a range of solute concentration. This difference can strongly affect the measured water flux and the predicted membrane performance, especially in the case of seawater-river water pairing, for which the draw side concentration is low. Accordingly, we recommend that the van ’t Hoff linear approach is not used to determine the osmotic pressure. In addition, the external mass transfer coefficients should not be neglected in predicting structural parameter, as is typical in the literature, unless their values are greater than approximately 8–10 × 10^−5^ m/s. Failing to consider these resistances can lead to the overestimation of the membrane structural parameter at typical cross-flow velocities of 20–35 cm/s in PRO and FO. Consequently, the accurate prediction of external mass transfer coefficients is crucially important for the accurate evaluation of the membrane properties.

## Figures and Tables

**Table 1:** Selective layer characteristics of the Porifera and NanoH_2_O membranes determined by cross-flow RO experiments at 22 °C and 0.20–0.25 m/s cross-flow velocity. deionized water (DI water) water was fed at 3, 5, 7, 9 bar to measure the *A* values, and 2 g/L NaCl solution was used to measure the *B* and *R* values at 3, 5, 7 bar.

Membrane	Water Permeability, *A*, 10^−7^ m/s·bar	Salt Permeability, *B* 10^−7^ m/s	NaCl Rejection Coefficient, %R
Porifera	5.0 ± 0.15	1.5 ± 0.6	90 ± 4.6
NanoH_2_O	1.0 ± 0.03	0.5± 0.12	90.2 ± 2.5

**Table 2:** General operating and transport parameters.

Parameters	Values
Feed solute concentration	0.015 M
Solute concentration in the draw solution	0.6 M, or varies
External mass transfer coefficients, draw side	3.85 × 10^−5^ m/s
External mass transfer coefficient, feed side	3.85 × 10^−5^ m/s
Diffusion coefficient at high salinity	1.367 × 10^−9^ m^2^/s
Diffusion coefficient at low salinity	1.294 × 10^−9^ m^2^/s
Hydraulic pressure difference	10 bar (or 0 and 15 bar)
Membrane transport parameters	*A* = 1.9 × 10^−7^ m/s·bar or varies *B* = 5.02 × 10^−7^ m/s or varies *S* = 5 × 10^−4^ m or varies

**Table 3:** Predicted values of the structural parameter for NanoH_2_O membrane as a function of draw salt concentration. DI is used for the feed phase; the osmotic pressure was predicted by the van ’t Hoff approach and by the OLI software for calculation of the structural parameter, *S*, with (*k_d_ = k_f_* = 2 × 10^−5^ m/s) and without external mass transfer resistances (*k_d_ = k_f_→*∞).

PRO						FO				
*C_d_*, mol/L, PRO, FO	*J_w_,* 10^−6^ m/s	*S*, 10^−6^ m NanoH_2_O				*J_w_*, 10^−6^ m/s	*S*, 10^−6^ m NanoH_2_O			
		van ’t Hoff		OLI *			van ’t Hoff		OLI *	
		1 *	2 *	1 *	2 *		1 *	2 *	1 *	2 *
0.27	0.28	15,950	15,788	14,540	14,364	0.26	8438	8350	7267	7178
0.62	0.37	15,396	15,235	14,610	14,430	0.32	9898	9812	9086	8999
1.03	0.40	16,687	16,531	16,270	16,049	0.47	7262	7179	6887	6804
1.53	0.47	15,510	15,355	15,306	15,222	0.52	7531	7448	7418	7335
Average		15,886	15,727	18,182	15,016		8282	8197	7665	7579

1 *: *k_d_ = k_f_→*∞; 2 *: *k_d_ = k_f_* = 2 × 10^−5^ m/s; OLI *: π = 5.94028*C*^2^ + 37.4521*C.*

**Table 4:** Predicted values of the structural parameter for the Porifera membrane as a function of draw salt concentration. DI is used for feed phase; the osmotic pressure was predicted by the van ’t Hoff approach and by the OLI software, using the curve-fit expression (π = 5.94028*C*^2^ + 37.4521*C* for *C* > 0.6) and also the linear interpolation of the OLI data given in Ref. [35], for calculation of the structural parameter, *S*, with (*k_d_ = k_f_* = 2 × 10^−5^ m/s) and without external mass transfer resistances (*k_d_ = k_f_*→∞).

Draw Concentration, M	*S* (van ’t Hoff), 10^−6^ m		*S* (OLI, Interpolated from Table), 10^−6^ m		*S* (OLI, Curve Fit), 10^−6^ m;	
	1 *	2 *	1 *	2 *	1 *	2 *
0.26	2057	1858	1976	1770	1815	1591
0.48	1370	893	1118	1327	1257	1034
0.50	1114	1169	1078	847	1019	766
0.60	1200	999	1169	962	1108	885
0.82	1250	1058	1228	1041	1191	1003
0.88	1049	856	1029	832	1002	799
1.28	1126	950	1163	989	1102	923
1.33	1055	877	1056	879	1150	973
Average:	1169	991	1135	1024	1138	936

1 * *k_d_ = k_f_*→∞; 2 * *k_d_ = k_f_* = 2 × 10^−5^ m/s.

**Table 5:** Application of measured data of Manickam and McCutcheon [40], for illustration of the effect of the external transfer coefficient on the predicted values of the structural parameter. The first two columns contain data published in [40]. The measured water flux data is shown to one decimal point in order to reflect that the reading error can reach 10% [40]. The third column (*k_d_ = k_f_*→∞) is data published in [40]. The fourth and fifth columns are calculations conducted in this study. (*A* = 0.606 L/m^2^h = 1.71 × 10^−7^ m/s·bar; *B* = 0.699 L/m^2^h = 1.94 × 10^−7^ m/s; *D* = 1.5 × 10^−9^ m^2^/s; *C_f_* = 0; Δ*P* = 0).

	*C_d_* mol/L	*J_w_,* Measured 10^−6^ m/s	*S*, (OLI Software) 10^−6^ m			*S*, (van ’t Hoff), 10^−6^ m	Error, %
			*k_d_ = k_f_→*∞	*k_d_ =* 5 × 10^−5^ m/s, *k_f_→*∞	*k_d_ =* 2 × 10^−5^ m/s, *k_f_→*∞	*k_d_ = k_f_→*∞	
P	0.5	2.8	791	649(*S/S*_∞_ = (0.82)	356 (0.45)	1117	29.1
R	1.0	4.9	803	708 (0.88)	514 (0.64)	897	10.5
O	1.5	6.4	789	726 (0.92)	584 (0.74)	816	3.3
F	0.5	2.1	327	298 (0.91)	252 (0.77)	459	28.8
O	1.0	3.0	434	404 (0.93)	360 (0.83)	491	11.6
	1.5	4.0	395	360 (0.92)	320 (0.81)	415	4.8

**Table 6:** Application of measured data of Tang et al. [37] to recalculate the effect of the measured water flux on the prediction of structural parameter, using OLI software instead of the van ’t Hoff linear approach, and to illustrate the effect of the external transfer coefficient on the predicted values of *S*. The first two columns (water flux, predicted structural parameter) are data published in [37], and the other data are calculated by authors of this paper. *A*–*S* indicates that it is less than zero, i.e., the measured water flux is higher than expected for the tested conditions. (*A* = 2.22 × 10^−7^ m/s·bar; *B* = 1.7 × 10^−7^ m/s; *D* = 1.5 × 10^−9^ m^2^/s; *C_f_* = 0.01 M; Δ*P* = 0).

	*C_d_* mol/L	*J_w_,* *Measured* 10^−6^ m/s	*S*, (van ‘t Hoff) 10^−6^ m *k_d_ = k_f_→*∞	*S*, (OLI Software), 10^−6^ m,		
				*k_d_ = k_f_→*∞	*k_d_ =* 5 × 10^−5^ m/s *k_f_*→∞	*k_d_ =* 2 × 10^−5^ m/s *k_f_*→∞
	0.5	4.2	581	210	-	-
P	1.0	7.3	546	474	320 (0.675)	-
R	2.0	11.6	471	481	407 (0.846)	142 (0.295)
O	3.0	13.6	431	499	447 (0.896)	332 (0.665)
	4.0	15.3	460	494	449 (0.91)	361 (0.730)
	0.25	1.6	467	227	197 (0.87)	152 (0.67)
F	0.75	3.0	460	381	352 (0.92)	309 (0.81)
O	1.5	4.2	460	441	412 (0.93)	369 (0.84)
	2.0	4.8	456	461	431 (0.93)	385 (0.84)
	3.0	5.6	456	490	458 (0.93)	410 (0.84)

## Nomenclature

*A*	water permeability, m/(s bar)
*B*	salt permeability, m/s
*C*	salt concentration, mol/L
*D*	fluid diffusion coefficient, m^2^/s
*E*	power density, W/m^2^
*J_s_*	solute transport rate, kg/m^2^s
*J_w_*	water flux, m/s
*J_w_*_,∞_	water flux at *k_d_* = *k_f_*→∞, m/s
*k*	diffusive mass transfer coefficient, m/s
*P*	hydraulic pressure, bar
*R*	rejection coefficient
*S*	structural parameter, m
*S*_∞_	structural parameter without external mass transfer resistances, m
*v*	convective fluid velocity perpendicular to the membrane surface, m/s
**Greek**	
β	diffusive plus convective transport coefficient, m/s
π	osmotic pressure, bar
δ	thickness of the fluid boundary layer, m
ε	porosity,
τ	tortuosity,
Δ*C_m_*	concentration difference on the active layer, mol/L
Δ*P*	hydraulic pressure difference, bar
Δ*π*	effective osmotic pressure difference on the active layer, bar
**Subscripts**	
b	bulk solute concentration
d	draw solution
f	feed solution
m	membrane active layer
i	interface between selective and the support layer (with *P*, *π*, β, or *C*)
p	permeate
s	support layer (with *S,* τ, ε, δ, or *D*)
sp	external interface of the support layer
w	water

## Appendix A. Concentration Distribution and Nomination of Transport Parameters in PRO and FO

The interface concentrations and the external mass transfer coefficients in the PRO and FO membrane processes are illustrated in Figure A1. The subscripts *d* and *f* correspond to the draw and the feed side, respectively. The interface concentrations of the asymmetric membrane are *C_m_, C_s_* and *C_sp_*; those of the fluid phase are *C_d_* and *C_f_*; and the diffusive mass transfer coefficients of the membrane transfer layers are *k_d_*, *B*, *k_sp_* and *k_f_,* while the corresponding convective ones are β*_d_*, β*_sp_*, β*_f_*.

## Appendix B. Osmotic Pressure as a Function of Draw Concentration

The osmotic pressure as a function of NaCl (left-hand vertical axis) and MgCl_2_ (right-hand vertical axis) solute concentrations is illustrated in Figure A2. Differences in the osmotic pressure values based on the van ’t Hoff linear approach and OLI Stream Analyzer [4,35,46] are shown. The deviation between the two osmotic pressure curves increases as a function of draw concentration, especially in the case of MgCl_2_. For example, the osmotic pressure difference between the van ’t Hoff and OLI data is about 284 bar at *C* = 3 M [4]. Though the difference is lower for NaCl, particularly when C < 2 M, it is still not negligible, as shown in this study.

## Appendix C. Estimation of the Uncertainty of Predicted Data

This study used an in-house computer program written in Quick-Basic computer language. Measured data from the literature were used to calculate the water flux and power density as a function of different membrane parameters. The error in measured water flux from the literature can reach up to 10%. We estimate a measurement error of 20% to ensure that the reading error does not increase the uncertainty of our calculations. Accuracy of our calculated water flux data is 16 decimals using Equations (7) and (11) for PRO and Equation (12) for FO; it is fitted to the measured data within 0.01% accuracy for all calculations of structural parameter. The structural parameter was iteratively calculated using Equations (16) or (17), and/or (18) and (19) until the error was less than 0.1%.

Uncertainty of the PRO and FO experimental data: every data point for both the water flux and the solute concentration was measured in triplicate; also, some of these triplicate measurements were repeated. The average values of data were then plotted in Figure 2; Figure 3. Generally, it can be stated that the scatter of these data reached the value of ±20%. The error of the results of RO experiments for determination of the solute permeability, applying triplicate measurements again, was obtained to be ±40%.
